# Systematic review and meta-analysis of recombinant herpes zoster vaccine in immunocompromised populations

**DOI:** 10.1371/journal.pone.0313889

**Published:** 2024-11-25

**Authors:** Fawziah Marra, Michael Yip, Jacquelyn J. Cragg, Nirma Khatri Vadlamudi

**Affiliations:** 1 Faculty of Pharmaceutical Sciences, University of British Columbia, Vancouver, BC, Canada; 2 Department of Pediatrics, Faculty of Medicine, University of British Columbia, Vancouver, BC, Canada; 3 School of Population and Public Health, Faculty of Medicine, University of British Columbia, Vancouver, BC, Canada; Beilinson Hospital: Rabin Medical Center, ISRAEL

## Abstract

**Importance:**

Herpes zoster infection is common in immunocompromised individuals. Recently, the Advisory Committee on Immunization Practices recommended immunizing with the recombinant zoster vaccine (RZV).

**Objective:**

To evaluate the efficacy, immunogenicity and safety of RZV in immunocompromised individuals, such as transplant recipients, cancer patients undergoing chemotherapy, individuals with preexisting autoimmune diseases and HIV-infected patients.

**Data sources and selection:**

From January 1984 to October 2023, a systematic search of PubMed, MEDLINE, EMBASE, Scopus, Web of Science, CINAHL, and Cochrane CENTRAL was performed. Randomized clinical trials (RCT) evaluating RZV compared to placebo in immunocompromised adults were selected.

**Data extraction:**

Study characteristics and estimates on the incidence of herpes zoster, immune responses, and safety data were extracted from studies. Estimates were pooled using random-effects meta-analysis. Differences by study-level characteristics were estimated using subgroup meta-analysis and metaregression.

**Results:**

Seven RCTs were included. Compared to placebo, RZV reduced the incidence of herpes zoster across all ages by 81% (RR: 0.19, 95%CI: 0.09, 0.44), with moderate heterogeneity across the studies (I2 = 60.49%; τ2 = 0.31; P = 0.07). RZV significantly increased humoral and cellular immunity one month after the last dose. Transplant and past malignancy were associated with lower immunogenicity. RZV was more reactogenic with more local and systemic adverse events. There was no difference in serious adverse events or death between the two arms.

**Conclusion:**

This study suggests that RZV reduces the risk of herpes zoster infection in immunocompromised individuals. This vaccine should be routinely offered to immunocompromised individuals, preferably before chemotherapy or treatment.

## Introduction

Primary infection with the varicella-zoster virus (VZV) or chickenpox during childhood leads to virus latency in the spinal and cranial sensory ganglia [1, 2]. Herpes zoster (HZ) infection or shingles, representing reactivation of VZV, is characterized by a unilateral, cutaneous, painful vesicular rash that typically presents in a single dermatome [3]. Postherpetic neuralgia (PHN), chronic neurologic pain that persists after the initial infection, is the most common and severe complication [3, 4]. In the USA, there are approximately 1 million cases of HZ each year, with 1 in 10 developing PHN [5]. Other complications of HZ include ophthalmitis, nerve palsies, neuromuscular disease (including Guillain-Barré syndrome), and secondary bacterial infections. [1]

The risk factors for reactivation are age and immunosuppression, either through disease or treatment, particularly T-cell-related suppression [6, 7]. This includes individuals with bone marrow or solid-organ transplants, lymphatic (leukemias and lymphomas) or other cancers, AIDS or HIV (particularly with CD4 counts less than 200/mm^3^), patients on daily prednisone of 20mg/day or more, immunomodulator therapies (>3mg/kg weekly of azathioprine; >1.5 mg/kg weekly mercaptopurine; and >0.4mg/kg weekly of methotrexate; any dose of tumour necrosis factor inhibitor), and those with cellular immune deficiency [8].

The incidence of HZ is 3–5 per 1000 person-years in the general population, but it can be as high as 31 per 1000 person-years in the immunocompromised [7, 9]. Herpes zoster rates are highest in individuals with hematopoietic stem cell transplant (HSCT) [10, 11] and hematologic malignancies [12, 13] with rates of approximately 30 per 1000 person-years. Solid organ transplant (SOT) recipients and those with solid tumor malignancies have rates ranging from 22 to 28 per 1000 person-years [14, 15]. Although HZ rates are lowest in people living with HIV on highly active antiretroviral therapy (ART), [16, 17] they are still 3 to 5 times above the general population at 10 cases per 1000 person-years [18, 19]. Although the risk of HZ is highest when their immunity is at the lowest point (e.g., CD4 counts less than 200/mm3), HZ can occur at any point in their disease course. Furthermore, recurrences and serious complications, such as disseminated disease, are more frequent [9, 20].

In 2006, a live attenuated vaccine (Zostavax) was licensed for immunocompetent adults aged ≥60 [21, 22]. Zostovax had an efficacy of approximately 50% for preventing HZ and 66% for postherpectic neuralgia (PHN) [23]. However, being a live vaccine meant that its use was contraindicated in immunocompromised individuals. A recombinant (non-live) adjuvanted zoster vaccine (RZV; Shingrix) was approved in 2017 for use in immunocompetent adults aged ≥50. The pre-licensure trials showed an efficacy for this vaccine at 97% that did not vary by age (>80 year old group had the same efficacy as the 50–59 year group) [24, 25]. One year later, this led to the preferential recommendation for the use of RZV over ZVL for immunocompetent adults over 50 years by a number of national immunization committees, including the National Advisory Committee on Immunization (NACI) in Canada [26] and Advisory Committee on Immunization Practices (ACIP) in the USA [27].

In July of 2021, the FDA and ACIP further expanded the indication for RZV to include those aged ≥19 who are or will be immunocompromised, either because of disease or receipt of drugs causing immunodeficiency [28, 29]. The main caveat was that optimally, RZV should be given before planned therapy with immunosuppressive drugs. This systematic review will evaluate the current literature on the use of RVZ compared to a placebo in immunocompromised populations. In addition, we will evaluate the overall efficacy of RZV in all immunocompromised populations.

## Materials and methods

This systematic review and meta-analysis were reported according to the PRISMA statement for randomized control trials (RCTs) [30, 31]. A pre-specified protocol that was developed before the literature review was followed.

### Search strategy

A systematic search was conducted using PubMed, MEDLINE, EMBASE, Scopus, Web of Science, CINAHL, Cochrane CENTRAL for articles from January 1984 to October 2023. Keywords and subject headings included herpes zoster, vaccination, immunization, recombinant vaccine, Shingrix, immunocompromised, autoimmune disease, transplantation, cancer, chemotherapy, radiation, steroids. There was no language restriction. A detailed search strategy can be found in the **S1 Table**. A manual search was conducted through a bibliography review of the included studies.

### Inclusion and exclusion

Studies were included if they were randomized controlled trials (RCT) evaluating recombinant herpes zoster vaccine in immunocompromised patients. Immunocompromised was defined as solid organ transplant patients, hematopoietic stem cell transplant patients, chemotherapy patients, HIV patients, or those on immunosuppressive therapy. Studies were excluded if they were review articles, meta-analyses, conference abstracts or observational studies. Studies evaluating herpes live vaccine and immunocompetent patients were also excluded. One independent researcher (MY) conducted the initial search with a librarian and the initial screening of the title and abstracts; full text review was conducted by two authors (MY and FL), with the third author adjudicating any discrepancies (JC).

### Data extraction

Two authors (MY/FL) independently completed the data extraction process, using a standardized form. Any disagreements were resolved through discussion and consensus with a third person (JC). Data were extracted on characteristics of the studies with author name, year of publication, title, case definitions, demographics such as age, comorbidities, race and main outcomes measured regarding efficacy and safety. Specific endpoints for efficacy included incidence of zoster, humoral and cell-mediated immunity, risk ratio associated with herpes zoster events, geometric mean concentration (GMC) ratio and geometric mean titer (GMT) ratio. Safety outcomes included solicited local (i.e., injection site pain, redness, and swelling) and systemic events (i.e., fever with temperature ≥37.5°C, headache, fatigue, nausea, vomiting, diarrhea and/or abdominal pain, myalgia and shivering) within 7 days post-vaccine, including grade 3 reactions. We also extracted any unsolicited adverse reaction that occurred within 30 days post-vaccine, including severe and fatalities.

### Outcome assessment

Studies that evaluated the efficacy of RZV against HZ used standard case definitions used in the pre-licensure study of immunocompetent persons 50 years of age and older [24]. A suspected case of HZ was defined as a new unilateral, dermatomal rash accompanied by pain or a vesicular rash suggestive of VZV infection and subsequently confirmed by PCR; or a clinical presentation and specific laboratory findings (PCR, culture, or immunohistochemical staining) specific to VZV infection in the absence of characteristic zoster-related rash.

Since there is no correlate of protection for HZ, investigators studied both humoral and cellular responses to RZV and compared them to placebo. Humoral immune responses included serum anti-glycoprotein E concentrations (anti-gE) while cellular immune responses included frequencies of gE and VZV-specific T cells expressing activation markers and gE-specific CD4[2+] T cells per 10^6^. The CD4+ T cells needed to express at least 2 of the following 4 activation markers: interferon-γ, interleukin-2, tumor necrosis factor α, and CD40 ligand. Immunogenicity was assessed in several ways. The geometric mean (GM) ratio represents the mean fold increase in geometric mean titers from pre‐vaccination to post-vaccination (taken one month after the last dose). Seroprotection rate for humoral immunity was defined as the percentage of participants with a postvaccination anti–glycoprotein E antibody concentration of at least 4-fold the prevaccination concentration. For cellular immunity, seroprotection was defined as the percentage of participants with postvaccination CD42+T-cell frequencies of at least 2-fold the threshold of 320 or at least 2-fold the prevaccination levels.

Pooled outcomes included HZ incidence, determined from cases occurring during the entire study period, and seroprotection rate at one month after the last dose, stratified by age (18–49 years, ≥50 years). Efficacy outcomes up to 12–15 months post‐vaccination were also assessed. Safety outcomes included solicited local and systemic events within 7 days post-vaccine, and any unsolicited adverse reaction within 30 days post-vaccine, including severe and fatalities.

### Quality assessment

The quality of included studies was assessed independently by two authors (JC/MY) according to Version 2 of the Cochrane Risk of Bias Tool (RoB 2) for randomized trials [32]. We assessed the possible risk of bias associated with five domains: the randomization process, deviations from intended intervention, missing outcome data, measurement of the outcome, and selection of the reported outcome. We recorded all answers to signaling questions and rated each domain as being “low risk,” “some concerns,” or “high risk,” as well as the overall risk. We generated ‘risk of bias graph’ **(S1 Fig)** and ‘risk of bias summary’ **(S2 Fig)** figures based on the overall risk of bias judgement.

### Statistical analysis

The risk ratio (RR) from individual studies was directly extracted or was calculated from the ratio of the proportion of herpes zoster events in vaccine group compared with placebo group. Since the prevalence of zoster infection is less than 10%, odds ratios (ORs) were considered comparable to RR [33]. The GM ratio and 95% confidence interval (CI) was used as the effect measure for comparing immunogenicity in the recombinant vaccine and placebo groups. The pooled proportions with 95% CI were calculated for those who reached the four-fold or two-fold post-vaccination titer threshold for humoral and cellular immunity, respectively for vaccine and placebo groups. If data were reported in figures only, we estimated the value using specialized software, PlotDigitizer^©^.

The heterogeneity of the included studies was assessed with the I-squared index, with a higher percent reflecting increasing heterogeneity; we assumed substantial heterogeneity when the I^2^ statistic was >50% [34]. In the presence of heterogeneity, pooled RRs were computed using a random‐effects model with inverse probability weighting, along with corresponding 95% confidence intervals. Lastly, we used funnel plots and Egger’s test to assess the presence of publication bias and small study bias for efficacy, immunogenicity and safety outcomes. When applicable, meta-regression were used to assess the heterogeneity in the true effects while accounting for study and population characteristics. All analyses were performed using R Statistical Software (v4.3.1; R Core Team 2021). The meta-analysis and forest plots were completed using the meta (version 6.5.0) and metafor (version 4.6.0) R packages.

## Results

**Fig 1:**
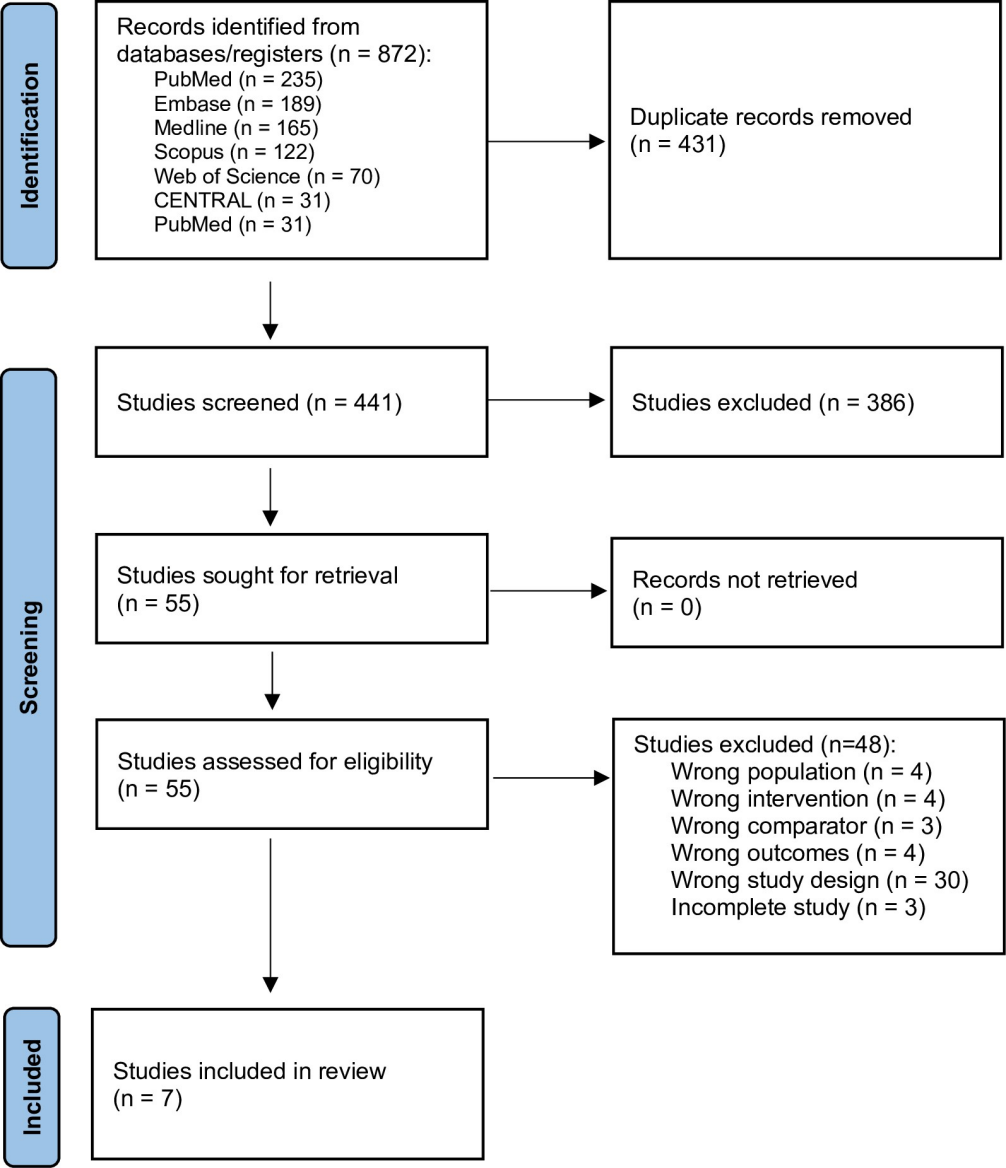
Flow diagram of study selection.

A total of 872 studies were initially identified; after removing duplicates, and evaluating titles and abstracts, 55 studies were included in the full text review, and seven studies were included in the meta-analysis **(Fig 1).**

### Trial characteristics

All seven studies were randomized, multicentre, placebo-controlled clinical trials. The populations studied included 2 studies on hematopoietic stem cell transplant recipients, [35, 36], one study each on the following: haematological malignancies, [37] solid-organ tumors renal [38], transplant recipients, [39], individuals with pre-existing immune-mediated diseases, [40] and HIV-infected individuals [41]. Two studies were phase 1/2 clinical trials designed to evaluate the safety and appropriate dosing schedule of RZV, including a 3-dose schedule; the remaining studies were phase 3 clinical trials that used a 2-dose RZV schedule. Three trials evaluated the vaccine’s efficacy on HZ, and all the trials except Dagnew et al. (2020) evaluated immunogenicity. For the secondary endpoints, only one trial evaluated efficacy against PHN, but all looked at adverse reactions. **Table 1** provides details of the included studies and the study population. **Table 2** shows the results of the primary and secondary outcomes in the seven clinical trials. Further details are provided in **S2 and S3 Tables**.

**Table 1:** Study characteristics.

Author, year	Design, Bias	Period	Patient population	Inclusion criteria	Exclusion criteria	Intervention	Primary Outcome^a^	Secondary Outcomes^b,c,d^
Stadtmauer, 2014	RCT, SB, PC, Phase 1/2a Bias = Some	2009 to 2012	Autologous HSCT	Age 18+ HSCT 50–70 days prior to first vaccination Hx of prior VZV infection Non-childbearing female	Allergy to vaccine component Prior HZV (any) Prior HZ case (1y) Experimental medication (30d) Acute disease or fever Received VZV since transplant Immunoglobulins or blood transfusion since transplant	1) 3 doses ge/AS01B 2) 3 doses gE/AS01E 3) 2 doses gE/AS01B 4) 3 doses placebo (0, 1, 3 mon or 0,3 mon)	Humoral response rate at 1 month after last vaccine dose	Humoral and Cellular response rate at months 1, 2, 3, and 15 post-vaccination GM ratio Adverse events
Bastidas, 2019	RCT, SB, PC, MC (28) Phase 3 Bias = Low	2012 to 2017	Autologous HSCT	Age 18+ HSCT 50–70 days prior to first vaccination Hx of prior VZV infection Non-childbearing female or on contraception	Allergy to vaccine component Prior HZV (1y) Prior HZ case (1y) HIV infection On antiviral therapy Experimental medication (30d) Other live vaccines Pregnant or lactating; planning to be pregnant	1) 2 doses ge/ASO1B 2) 2 doses placebo (0, 1–2 mon)	HZ incidence rate ratio 1 month after last vaccine dose and various time points till study end	Humoral and cellular response rate at 1 month after last vaccine dose GM ratio Adverse events
Dagnew, 2019	RCT, SB, PC, MC (21) Phase 3 Bias = Low	2013 to 2015	Hematological malignancies	Age 18+ On therapy with immunosuppressives Non-childbearing female or on contraception	Allergy to vaccine component Prior HZV (1y) Prior HZ case (1y) HIV infection Experimental medication (30d) Other live vaccines CLL treated with only oral chemotherapy Scheduled HSCT during study period or HSCT (50d) Previously vaccinated against VZV	1) 2 doses ge/ASO1B 2) 2 doses placebo (0, 1–2 mon)	Post-hoc HZ incidence one month after last vaccine dose till study end^e^	Humoral and cellular response rate at 1 month after last vaccine dose GM ratio Adverse events
Dagnew, 2020	RCT, DB, PC, MC (18) Post-hoc of ZOE-50 and ZOE-70 studies Bias = Low	2010 to 2015	Potential immune-mediated disease (pIMD)f	Age 50+ Non-childbearing female or on contraception	Allergy to vaccine component Prior HZV (any time) Prior HZ case (any time) Receipt of immunosuppressants or immune modifying drugs for >15 consecutive days within 6 months prior to the first vaccine dose HIV infection Experimental medication (30d) Other live vaccines Enrolled in another trial Acute disease or fever Immunoglobulins or blood transfusion Previously vaccinated against VZV Significant underlying illness expected to prevent completion of study Pregnant or lactating; planning to be pregnant	1) 2 doses ge/ASO1B 2) 2 doses placebo (0, 2 mon)	HZ incidence rate ratio 1 month after last vaccine dose and various time points till study end	Adverse events
Vink, 2019	RCT, SB, PC, MC (6) Phase 2/3 Bias = Low	2013 to 2016	Solid organ tumor	Age 18+ Diagnosed with 1 or more solid organ tumor Receiving cytotoxic or immunosuppressive chemotherapy	Allergy to vaccine component Prior HZV (1y) Prior HZ case (1y) Other live vaccines Receiving target therapies without immunosuppressives Received systemic corticosteroids for more than 14 days consecutively (1m), or had previous chemotherapy ending (28d) Previously vaccinated against VZV (12m)	1) 2 doses ge/ASO1B 2) 2 doses placebo (0, 1–2 mon)	Humoral response rate at 1 month after last vaccine dose^g^	CMI response rate at 1 month after last vaccine dose GM ratio Adverse events
Vink, 2020	RCT, SB, PC, MC (9) Phase 3 Bias = Low	2014 to 2017	Renal transplant	Age 18+ Received ABO compatible allogenic renal transplant No allograft rejection (3 mon) Stable renal function Between 4 and 18 months after renal transplant	Allergy to vaccine component Prior HZV (1y) Prior HZ case (1y) Pregnant or lactating Other live vaccines Previously vaccinated against VZV (12m) Acute disease or fever Primary renal disease Prior allograft loss Multiple organs transplanted Evidence of significant proteinuria Any other autoimmune or pIMD Immunosuppressants for renal transplant (9m)	1) 2 doses ge/ASO1B 2) 2 doses placebo (0, 1–2 mon)	Humoral response rate at 1 month after last vaccine dose	CMI response rate at 1 month after last vaccine dose GM ratio Adverse events
Berkowitz, 2014	RCT, SB, PC, MC (3), Phase 1/2a Bias = Low	2010 to 2013	HIV patients	Age 18+ Non-childbearing female or on contraception If on ART, ≥50 cells/mm^3^ and HIV RNA <40 copies/mL If ART-naïve, then ≥500 cells/mm^3^ and HIV RNA ≥1000 copies/mL and ≤100,000 copies/mL	Allergy to vaccine component Prior HZV (1y) Prior HZ case (1y) Pregnant or lactating Other live vaccines Acute disease or fever Immunoglobulin or blood transfusion AIDS defining condition or malignancy or opportunistic infection (1y) Change in anti-retroviral regimen (3m) Immunosuppression from anything other than HIV or therapy Chronic immunosuppressive drugs (6m) Active HBV or HCV infection Concurrent HIV fusion inhibitors, CCR5 inhibitors or IL-2/IL-7/interferon Abnormal laboratory values from blood samples at screening	1) 3 doses ge/AS01B 2) 3 doses placebo (0, 2, 6 mon)	Humoral response rate at 1 month after last vaccine dose	CMI response rate at 1 month after last vaccine dose GM ratio Adverse events

RCT–randomised controlled trial; SB–single-blind; PC–placebo-controlled; MC–multicenter (# of countries); HSCT–hematologous stem cell transplant; VZV–varicella zoster virus; GM ratio–geometric mean ratio; anti-gE–anti glycoprotein E; Tx–transplant; NHL Non-Hodgkin B-cell lymphoma; CLL—chronic lymphocytic leukaemia; gE/AS01B VZV gE (50 mg)—AS01B liposome-based adjuvant: 50 mg monophosphoryl lipid A (MPL) and 50 mg Quillaja saponaria Molina 21 (QS21); gE/AS01E VZV gE (50 mg)—AS01E liposome-based adjuvant: 25 mg monophosphoryl lipid A (MPL) and 25 mg Quillaja saponaria Molina 21 (QS21); HIV–Human Immunodefiency Virus; HBV–Hepatitis B Virus; HCV–Hepatitis C Virus; ART–antiretroviral therapy

Footnotes

a Humoral response rate was defined as the percentage of participants with a postvaccination anti–glycoprotein E antibody concentration [anti-gE antibody] of at least 4-fold the cutoff (for participants with concentrations initially below the cut off) or at least 4-fold the prevaccination concentration (for participants with concentrations initially above the cutoff)

b The cell-mediated immunity (CMI) vaccine response rate was defined as the percentage of participants with postvaccination CD42+T-cell frequencies of at least 2-fold the threshold of 320, CD42+T cells per 10^6^ total CD4 T cells (for participants with concentrations initially below this threshold) or at least 2-fold the prevaccination CD42+T-cell frequencies (for participants with concentrations initially above this threshold).

c Adjusted geometric mean ratio (vaccine over placebo) for anti-gE antibody 95% CI LL is greater than 3 and for gE-specific CD4[2+] T cell frequencies is over one at one-month after second dose

d Adverse events recorded as solicited local reactions (e.g., pain, redness, swelling at the injection site), systemic reactions (e.g., fever, headache, fatigue, myalgia) on diary cards for 7 days after each vaccination. Intensity of the solicited reactions was scored on a 0 to 3 rating scale. Unsolicited adverse events (AEs) were reported from the study start to 30 days after the last vaccination. Serious adverse events and fatalities were recorded from time of first dose receipt to study end

e Primary outcome excluded participants with non-Hodgkin lymphoma and chronic lymphocytic leukemia

f Psoriasis, spondyloarthropathy, rheumatoid arthritis and celiac disease

g Primary outcome was in patients who received first vaccine dose 8 to 30 days prior to receipt of chemotherapy (Pre-Chemo)

**Table 2:** Primary outcomes.

Author, year	Sample size (per group)	Mean Age (years) Gender (%) Ethnicity (%)	Cumulative follow-up	Number of events	Incidence Vaccine efficacy % (95% CI)	Percentage of patients with a response rate to anti-gE humoral immune, % (95% CI)	Adjusted GM ratio for anti-gE Ab concentrations (95% CI)	Percentage of patients with a response rate to cell-mediated immunity, % (95% CI)	GM ratio for anti-gE CD4[2+] T cell concentration (95% CI)
Stadtmauer, 2014	Total 121, 1) 3 doses ge/AS01B N = 30 2) 3 doses gE/AS01E N = 29 3) 2 doses gE/AS01B N = 31 4) 3 doses placebo N = 30	59.0 (Range, 20–70) 35.0% female 83.3% white	15 mon	----	----	1) 3 doses ge/AS01B: 72.5% (53.0–82.5) 2) 3 doses gE/AS01E: 73.3% (55.4–88.6) 3) 2 doses gE/AS01B: 77.0% (56.5–91.0) 4) 3 doses placebo: 0.0% (0.0–13.3)	1) 3 doses ge/AS01B: 74.41 (25.74–215.09) 2) 3 doses gE/AS01E: 52.11 (17.36–156.41) 3) 2 doses gE/AS01B: 42.2 (16.07–110.82)	1) 3 doses ge/AS01B 100% (87.0–100) 2) 3 doses gE/AS01E 87.6% (67.6–97.5) 3) 2 doses gE/AS01B 76.0% (56.0–90.5) 4) 3 doses placebo 20.8% (7.3–42.3)	1) 3 doses ge/AS01B: 32.31 (17.78–58.71) 2) 3 doses gE/AS01E: 16.49 (8.87–30.68) 3) 2 doses gE/AS01B: 9.51 (5.23–17.32)
Bastidas, 2019	Total 1846, RZV: 922 PLB: 924	55.0 ±11.6 37.3% female	RZV: 1633 person years PLB: 1432 person years 24 mon	RZV: 49/870 PLB: 135/851	IRR 0.32 (0.22–0.44) VE: 68% (56–78)	RZV: 67% (56.0–77.0) PLB: 0% (0.0–4.7)	29.1 (5.2–309.0)	RZV: 93% (80.0–99.0) PLB: 0% (0.0–8.6)	29.0 (1.9–27.6)
Dagnew, 2019	Total 606, RZV: 286 PLB: 283	57.3 ±15.2 40.6% female 68.3% white	15 mon	RZV: 2/259 PLB: 14/257	IRR 0.14 (0.03–0.62) RZV: HZ incidence 8.5 per 1000-person years PLB: HZ incidence 66.2 per 1000-person years VE: 87.2% (44.3–98.6)	RZV: 80.4% (73.1–86.5) PLB: 0.8% (0.0–4.2)	29.75 (21.09–41.96)	RZV: 83.7% (69.3–93.2) PLB: 6.8% (1.4–18.7)	83.6 (69.4–93.1)
Dagnew, 2020	Total 1943, RZV: 983 PLB: 960	69.1 ±9.5 60.4% female 85.4% white	RZV: 3611.7 person years PLB: 3408.8 person years 24 mon	RZV: 4/936 PLB: 38/923	IRR 0.10 (0.01–0.64) RZV: HZ incidence 1.1 per 1000-person years PLB: HZ incidence 11.1 per 1000-person years VE: 90.5% (73.5–97.5)	----	----	----	----
Vink, 2019	Total 262, RZV: 130 PLB: 132	57.8 ±11.3 60.0% female 77.6% white	15 mon	----	----	Pre-Chemo RZV: 93.8% (85.0–98.3) PLB: 0.0% (0–3.6) Pre- & On-Chemo RZV: 86.1% (77–92.7) PLB: 0% (0–3.6)	Pre-chemo: 23.3 (17.9–30.0) Pre- & On-Chemo: 14.4 (10.7–19.5)	Pre-Chemo RZV: 50.0% (28.2–71.8) PLB: 0% (0–12.8) Pre- & On-Chemo RZV: N/A PLB: N/A	Pre-Chemo: 9.94 (3.63–27.19) Pre- & On-Chemo: N/A
Vink, 2020	Total 264, RZV: 132 PLB: 132	52.3 ±12.7 30.0% female 88.4% white	15 mon	----	----	RZV: 80.2% (71.9–86.9) PLB: 4.2% (1.4–9.5)	14.0 (10.9–17.9)	RZV: 71.4% (51.3–86.8) PLB: 0% (0–12.4)	17.26 (5.9–50.36)
Berkovitz, 2014	Total 123, RZV: 74 PLB: 49	46.0 ±10.9 5.7% female 87.8% white	18 mon	----	----	RZV: 96.2 (87–99.5) PLB: 2.8% (0.1–14.5)	46.22 (33.63–63.53)	RZV: 90.0% (68.3–98.8) PLB: 16.7% (3.6–41.4)	21.95 (12.97–38.02)

RZV–recombinant zoster vaccine; PLB–placebo; GM ratio–geometric mean ratio; anti-gE–anti glycoprotein E Antibody Concentration; IRR–incidence rate ratio; Pre-Chemo–Before chemotherapy

### Prevention against herpes zoster

**Fig 2:**
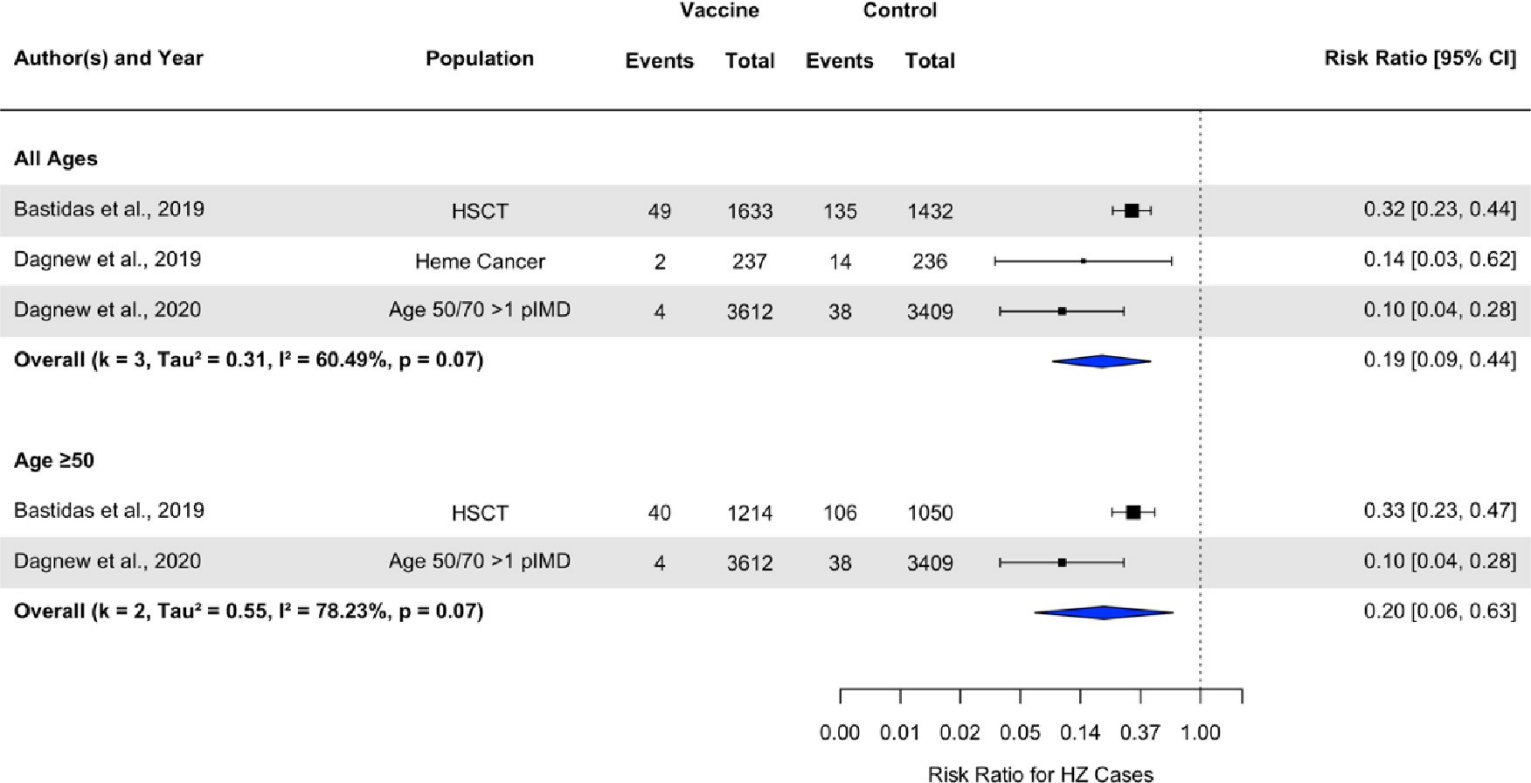
Forest plot of the efficacy of recombinant herpes zoster vaccine on herpes zoster incidence.

The forest plot (**Fig 2)** shows the results of the meta-analysis from the three studies that evaluated the incidence of herpes zoster following RZV compared to placebo. Herpes zoster incidence after two doses was significantly lower in the vaccine group compared with placebo (pooled RR: 0.19, 95% CI: 0.09, 0.44, I^2^ = 60.4%) among all adults. Similarly, lower risk of HZ incidence was observed among vaccine participants in comparison to controls in population ≥50 years of age (pooled RR: 0.20, 95% CI: 0.12, 0.36, I^2^ = 78.2%). Vaccine effectiveness was the highest for individuals who were least immunosuppressed which was participants with pre-existing potential immune-mediated diseases [38] and lowest for those categorized as HSCT recipients [36].

**S2 Table** shows that of the three studies, only Bastidas et al. [36] evaluated the efficacy of RZV against post-herpetic neuralgia, therefore we were unable to estimate a pooled outcome. It also shows the incidence of HZ at the end of the follow-up period (approximately one year after the last dose), which remained significantly lower in the vaccine arm compare to placebo (pooled RR: 0.19, 95%CI: 0.09, 0.40, I^2^ = 51.2%) (**S3 Fig).**

### Immunogenicity

**Fig 3:**
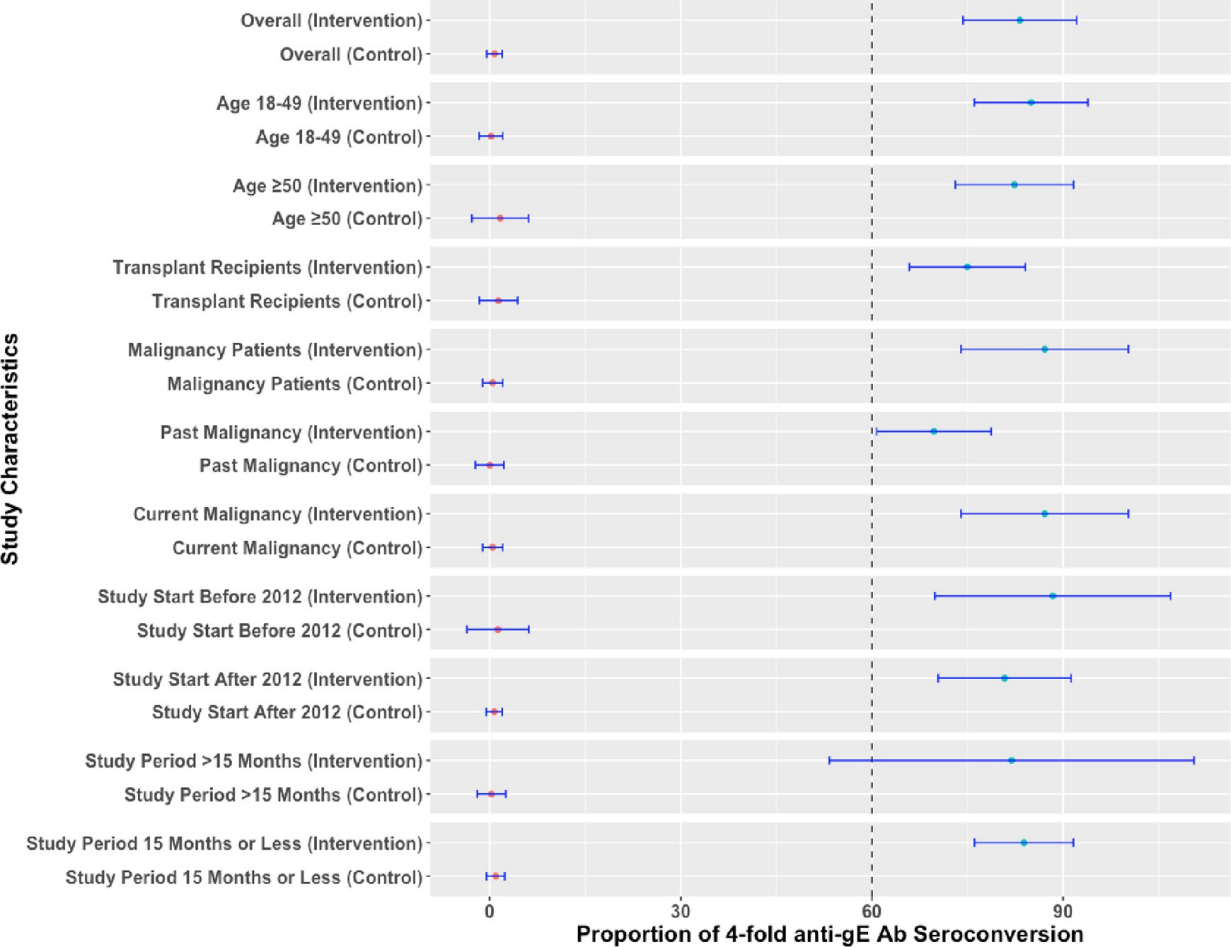
Pooled humoral immunogenicity for recombinant herpes zoster vaccine compared with control arm at one month after the last dose.

**Fig 4:**
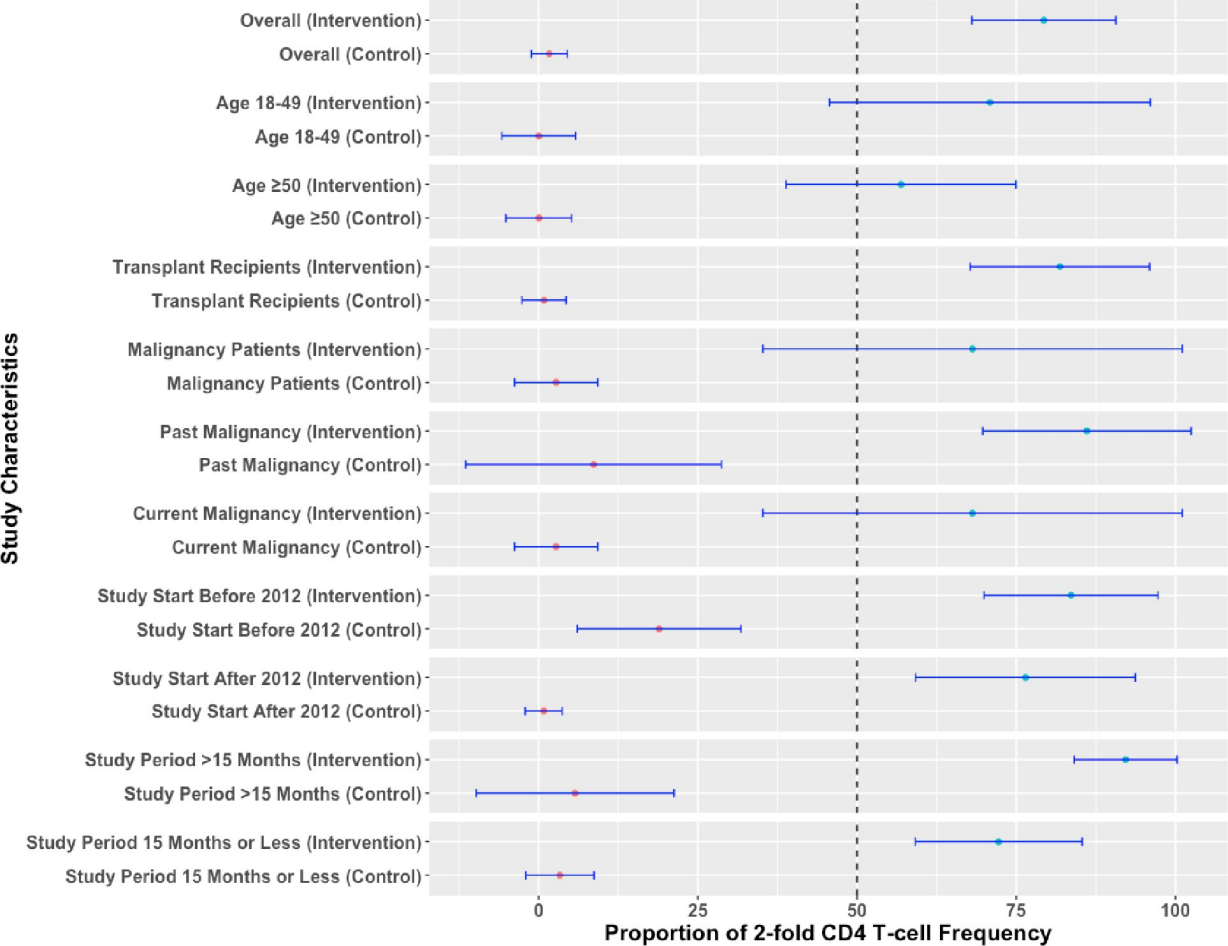
Pooled cellular immunogenicity for recombinant herpes zoster vaccine compared with control arm at one month after the last dose.

Meta‐analyses of six studies evaluating humoral and cellular immunogenicity at one month after the last dose are shown in **Figs 3 and 4**, respectively. For humoral immunity, the proportion of individuals with 4-fold anti-gE antibody seroconversion at 1 month after last dose was observed in 83.21% of vaccine group (95% CI: 74.31, 92.11) and it was significantly higher than the controls (0.74%; 95% CI: -0.48, 1.96). For cellular immunity, the proportion of individuals with 2-fold CD4 T-cell increase in frequency was noted among 79.33% of the vaccine group (95% CI: 68.02, 90.54) and it was substantially higher compared with the control group (1.65%; 95% CI: -1.18, 4.48).

We did not note any significant difference in efficacy for adults aged 50 and above compared with all ages in the meta‐regression and sub-group analyses **(Figs 2–4)**. Furthermore, transplant and past malignancy were associated with lower antibody response and seroconversion rates in sub-group analyses **(Figs 3 and 4).** No other study‐level variables had a significant impact on the immune response.

Similar results were seen with humoral (**S2 Table and S4 Fig)** and cellular immunity (**S2 Table and S5 Fig)** at the end of the follow-up period, which varied from month 13 to 18the proportion of individuals with increased humoral and cellular immunity remained significantly elevated in the vaccine arm compared to the placebo arms.

### Vaccine safety

**Fig 5:**
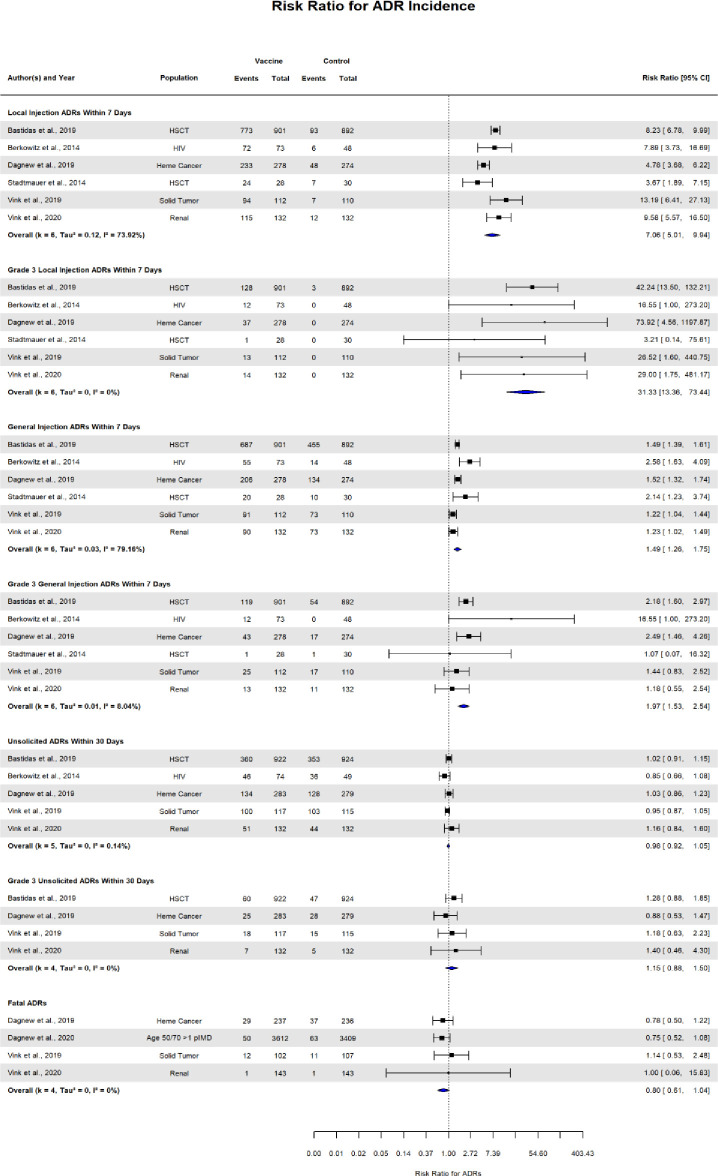
Forest plot of the effect of recombinant herpes vaccine compared with control arm on local and general adverse effects.

The table (**S4 Table)** shows the results of the data from the clinical trials on adverse events, as well as the RRs and 95% confidence intervals. A pairwise meta-analysis, shown in **Fig 5**, identified patients vaccinated with RZV had a higher rate of local injection adverse events within seven days (pooled RR: 7.06, 95% CI: 5.01–9.94), grade 3 local injection adverse events within seven days (pooled RR: 31.33, 95% CI: 13.36–73.44), general injection adverse events within seven days (pooled RR: 1.49, 95% CI: 1.26–1.75), and grade 3 general injection adverse events within seven days (pooled RR: 1.97, 95% CI: 1.53–2.54).

There was no statistical difference between RZV and placebo with regard to unsolicited 30-day adverse events (pooled RR: 0.98; 95% CI: 0.92–1.05), grade 3 unsolicited 30-day adverse events (pooled RR: 1.15; 95% CI: 0.88–1.50), and fatal adverse events (pooled RR: 0.88 95% CI: 0.61–1.04).

### Quality assessment

Although six of seven studies were considered low of risk when evaluated using Cochrane Risk of Bias Tool, Stadtmauer et al. [35] shows some concerns regarding missing outcome data as shown in **S1 Fig**. A funnel plot of studies that reported on herpes zoster incidence and immune response is presented in **S6 Fig**. The Egger’s test indicated the absence of significant publication bias.

## Discussion

This systematic review and meta-analysis identified protective and immunogenic effects of herpes zoster vaccine in immunocompromised populations, ranging from immune-mediated diseases to HIV-positive individuals. Our study findings suggest that RZV is superior to placebo for prevention of HZ with an 81% reduction compared to placebo; however, it should be noted that the pooled estimate is based on only three studies [36– 38]. Although we were not able to pool the data, Bastidas et al. showed a reduction in the incidence of PHN in their clinical trial–the only one that evaluated this endpoint of the seven trials [36].

Our meta-analysis showed that RZV produces significantly better immune responses, both in terms of humoral and cellular immunity, compared to placebo at multiple time points, including one month after the second dose and approximately 12 to 15 months post second dose. Better protection against HZ was seen in individuals who were less immunocompromised than those heavily immunosuppressed. Hematogenous stem cell transplant recipients are arguably the most immunosuppressed of the populations studied in this meta-analysis. Although vaccine efficacy against HZ of 68%, shown in the Bastidas trial, is lower than the 97% seen in non-immunosuppressed persons, it is similar to that of a heat-activated varicella zoster virus vaccine administered as a 4-dose schedule to HSCT patients [42]. This latter vaccine was administered 1 month pre-transplantation but posed logistical challenges with the large number of doses. As such, the 2-dose vaccine schedule would likely allow for a higher vaccine uptake rate in the immunocompromised populations. Dagnew and colleagues reported a higher efficacy against HZ of 87% [37] and 90% [38] in their study populations who had hematological malignancies and immune-mediated disease, respectively. Their findings are comparable to the pooled ZOE-50/70 data [25] of their whole population that showed RZV efficacy is maintained at >90% even in the oldest age group (>80 year-olds). The authors concluded that RZV showed a robust immune response in patients with preexisting immune conditions. But one has to keep in mind the limitations: this was a *posthoc* analysis that did not control for a type 1 error, the original clinical trials were not powered to look at this subset of patients, technically these are not immunocompromised persons as persons with pre-existing immune conditions who were undergoing immunosuppressive treatment were not included in the ZOE50/ZOE-70 studies.

Recent end-of-trial data show a 79.7% (95% CI 73.7–84.6) cumulative efficacy in immunocompetent participants aged 50 years and over, within the period from year six to year 11 after vaccination, demonstrating prolonged duration of protection that that patient population [43]. Our pooled analysis showed that RZV was highly immunogenic in our immunocompromised population, as shown by high humoral and cellular immune responses one month after the last dose compared to the placebo arm. Although these remained elevated at one year after receipt of the second vaccine dose of the vaccine, long term data on waning of immunity over time are needed for this population.

The phenomenon of immunosuppressed patients generating a better humoral and cellular immune response when the vaccine was administered pre-transplant or pre-chemotherapy was seen in a number of studies. This has prompted ACIP to recommend administering the vaccine before patients are heavily immunosuppressed if possible [29]. In SOT recipients, the recommendation is pre-transplant and, if not possible, then 6–12 months post-transplant when the graft is stable and patient is on minimal immunosuppression [29, 37]. Similarly, for cancer patients, administer RZV prior to chemotherapy, immunosuppressive mediations and/or radiation therapy [29, 39]. For patients receiving anti-B cell therapies (e.g., rituximab), administer RZV approximately 4 weeks prior to treatment [29, 39]. Patients with autoimmune conditions and immune system disorders should receive RZV when their disease is well controlled, that is, not during an acute flare-up, and if possible, it should be initiated prior to being placed on immunosuppressive medications [29, 38]. Receipt of RZV is tied to antiviral therapy for HSCT recipients who often get recurrent HZ infections and are placed on prophylaxis with antivirals (e.g., valacylovir or famciclovir). For these patients, RZV should be given 2 months prior to stopping antivirals, and 3–12 months after transplantation [29, 36]. Although our study showed RZV elicited a four-fold anti-gE response and 2-fold gE-specific CD4[2+] T cell response, it should be noted that a correlate of protection is not yet available for HZ.

Our study identified that patients vaccinated with RZV had a higher rate of local and general adverse events within seven days of vaccine receipt compared to placebo, many of these being at a grade 3 level. We did not see a statistical difference between RZV and placebo with regard to unsolicited 30-day adverse events, general or grade 3-related or fatal adverse events. The individual studies of RZV in immunocompromised patients that evaluated vaccine safety compared to placebo showed that RZV produced mild local reactions, with pain at the injection site being the most common side effect. Common systemic adverse events lasted approximately 3 days and included fatigue, fever and chills. Local grade 3 reactions occurred in 10.7% to 14.2% of RZV recipients, and systemic grade 3 reactions occurred in 9.9% to 22.3% of RZV recipients, compared with 0% to 0.3% and 6.0% to 15.5%, respectively, among placebo recipients. No evidence of graft-versus-host disease or allograft rejection was noted by Bastidas and colleagues [36, 44] nor was there an increased risk of rejection in renal transplant recipients who received RZV compared to placebo [37, 43]. Finally, increased disease flare-ups in patients with immune-mediated diseases or HIV-infected patients were not seen in greater numbers in the RZV group compared to placebo [38, 41, 43].

Two network meta-analyses have investigated the efficacy and effectiveness against prevention of HZ, comparing RZV and the live zoster vaccine [45, 46]. Both studies included randomized control trials, as well as observational studies in immunocompetent and immunocompromised patients. Our findings were consistent with the results seen among immunocompromised subjects wherein RZV was superior to placebo in prevention of HZ, with a risk ratio of approximately 0.30 (RCT data) and 0.35 (OBS data) [45]. For immunocompetent individuals, in comparison to the live zoster vaccine, individuals on RZV showed improved effectiveness against HZ, although reactogenicity was significantly higher with RZV. While these two metanalysis included a broader population than ours, with both immunocompetent and immunocompromised individuals included, this is also a limitation as there was much heterogeneity in the included studies and population, which was further enhanced by including both clinical trials and observational studies. To limit the heterogeneity, we chose to only include one type of study population—immunocompromised—and even with that we have a fairly heterogeneous population with severely immunocompromised patients, such as those who had undergone HSCT to those less immunocompromised with preexisting immunosuppressive diseases. We also decided not to include observational studies in our meta-analysis, of which there are two that evaluated real world evidence of RZV in those with autoimmune conditions [47, 48]. Our decision to not include the two studies is because it would not have produced meaningful results but should be explored when more studies are published. Another limitation of our meta-analysis is the small sample size of some of the studies, despite pooling of data. Both of these limits the generalizability of the results and should be viewed with caution.

## Conclusion

The non-live-virus recombinant zoster vaccine is efficacious, immunogenic and safe for immunocompromised persons 18 years of age and older as a 2-dose schedule. Administering RZV to highly immunocompromised persons whose risk for HZ is over ten times the general population would markedly diminish the incidence of this debilitating and often recurrent condition. ACIP recommends a 2-dose schedule for all immunocompromised populations, including HIV-infected persons [29]. Data from our meta-analysis and individual trials suggest the vaccine should be administered prechemotherapy, transplant or immunosuppressive medications, if possible. Although a 2-dose schedule means higher compliance and vaccine uptake need to be stressed to patients, ongoing long-term studies are needed to see the waning of immune responses and if booster doses may be necessary in this highly susceptible population further down the road.

## Supporting information

S1 Fig Summary assessment of quality of trials (risk of bias) using the Cochrane collaboration’s tool. Low risk, some concerns.
(TIF)

S2 Fig Individual assessment of quality of trials (risk of bias) using the Cochrane collaboration’s tool. Low risk, some concerns.
(TIF)

S3 Fig Pooled risk ratio for herpes zoster incidence at the 13-month follow-up. Long-term data on herpes zoster incidence with the vaccine and control arms.
(TIF)

S4 Fig Pooled humoral immunogenicity for recombinant herpes zoster vaccine compared with control arm at end of follow-up period. Long-term data on humoral immunogenicity with the vaccine and control arms.
(TIF)

S5 Fig Pooled cellular immunogenicity for recombinant herpes zoster vaccine compared with control arm at end of follow-up period. Long-term data on cellular immunogenicity with the vaccine and control arms.
(TIF)

S6 Fig Funnel plots to identify bias for herpes zoster incidence, humoral and cellular immunogenicity. Visualization of bias associated with the included studies.
(TIF)

S1 Table Search strategies. All studies identified in the literature search.
(DOCX)

S2 Table Results for secondary outcomes at end of follow up period. RZV–recombinant zoster vaccine; PLB–placebo; GM ratio–geometric mean ratio; anti-gE–anti glycoprotein E Antibody Concentration; IRR–incidence rate ratio; Pre-Chemo–Before chemotherapy.
(DOCX)

S3 Table Narrative of the included studies. Details of the seven studies included in this review.
(DOCX)

S4 Table Results for adverse drug reactions. RZV–recombinant zoster vaccine; PLB–placebo; ADRs–adverse drug reactions; RR–risk ratio.
(DOCX)

S5 Table Excluded studies. All studies that were excluded from the analyses, and reason(s) for exclusion.
(DOCX)

S6 Table Extraction table. All data extracted from the primary research sources for the systematic review and/or meta-analysis. The table includes the following information for each study: name of data extractors and date of data extraction, confirmation that the study was eligible to be included in the review.
(XLSX)
