# Supplementary figures and images for: Systematic review and meta-analysis of recombinant herpes zoster vaccine in immunocompromised populations
Source: PLoS One. 2024 Nov 25;19(11):e0313889. doi: 10.1371/journal.pone.0313889 (PMC11588208; doi:10.1371/journal.pone.0313889)

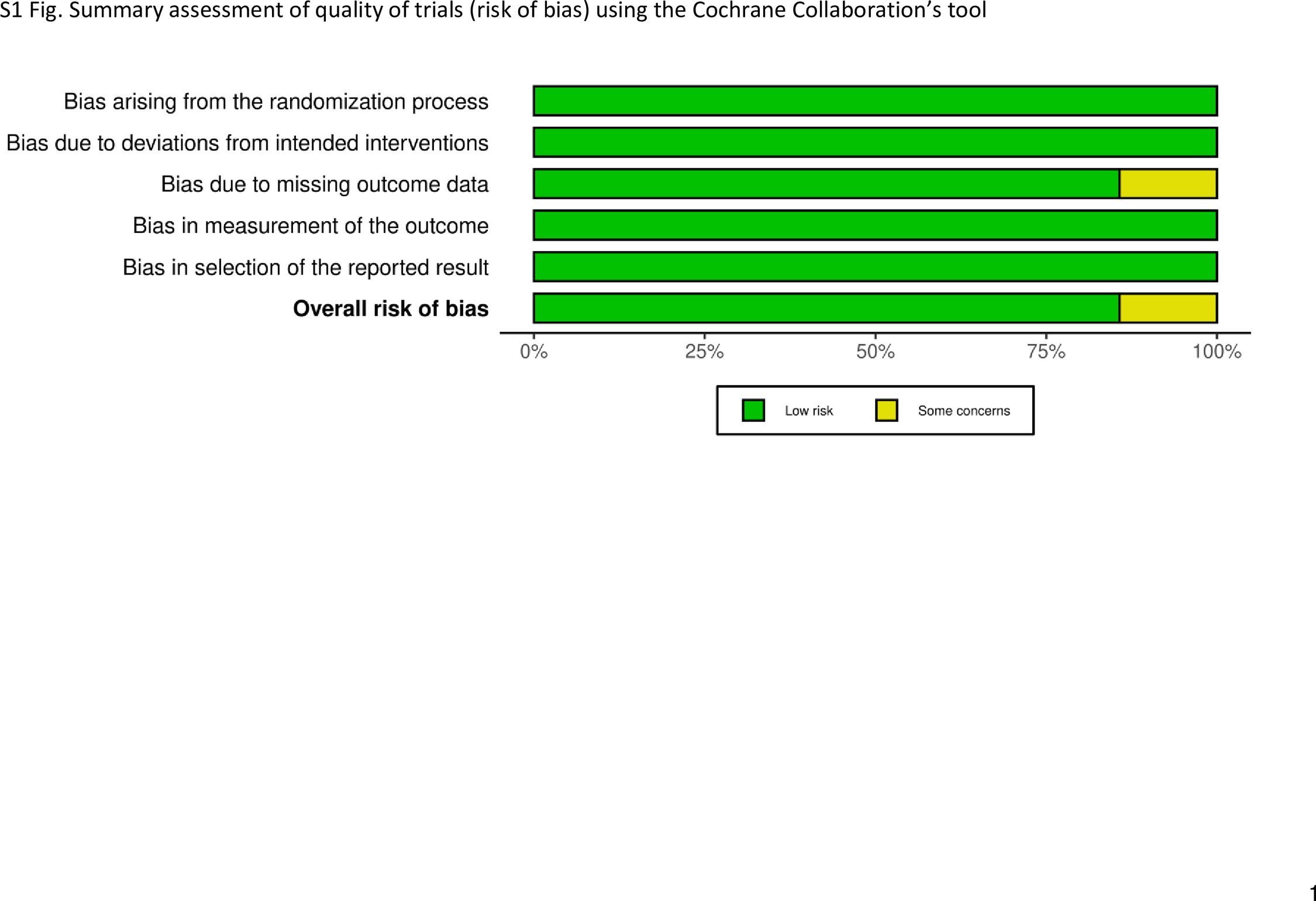

Supplement: S1 Fig — Low risk, some concerns. (TIF) [file pone.0313889.s001.tif]

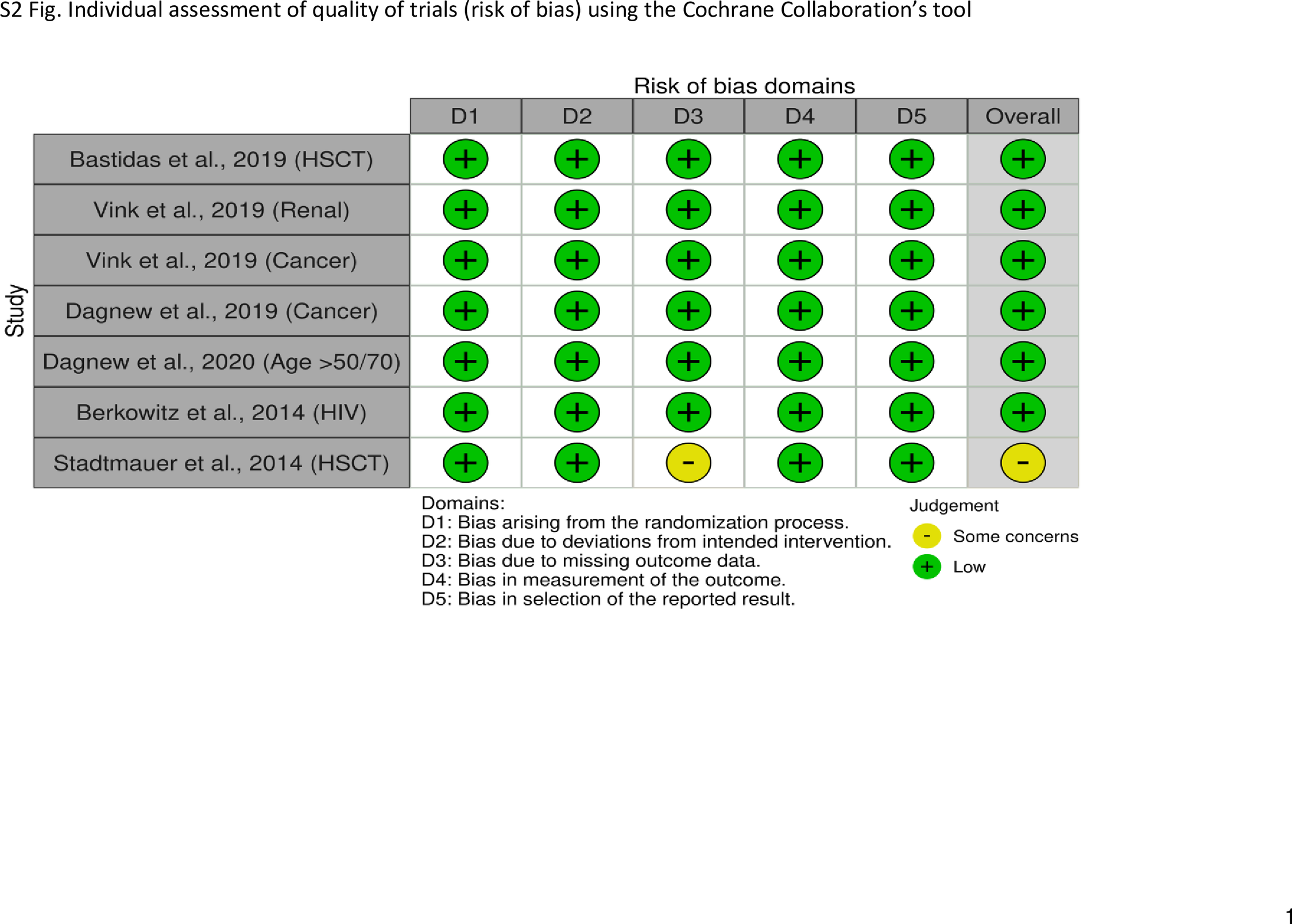

Supplement: S2 Fig — Low risk, some concerns. (TIF) [file pone.0313889.s002.tif]

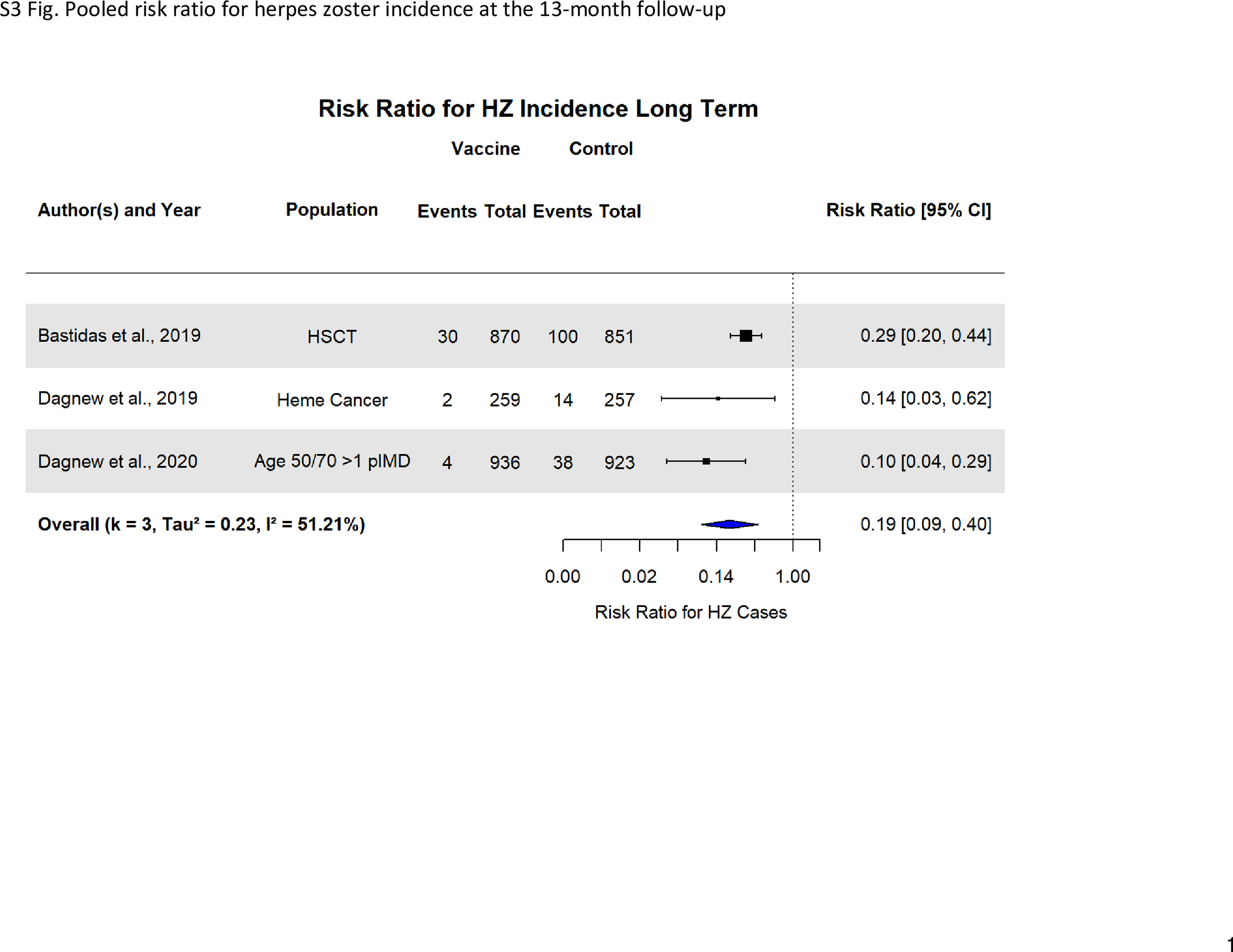

Supplement: S3 Fig — Long-term data on herpes zoster incidence with the vaccine and control arms. (TIF) [file pone.0313889.s003.tif]

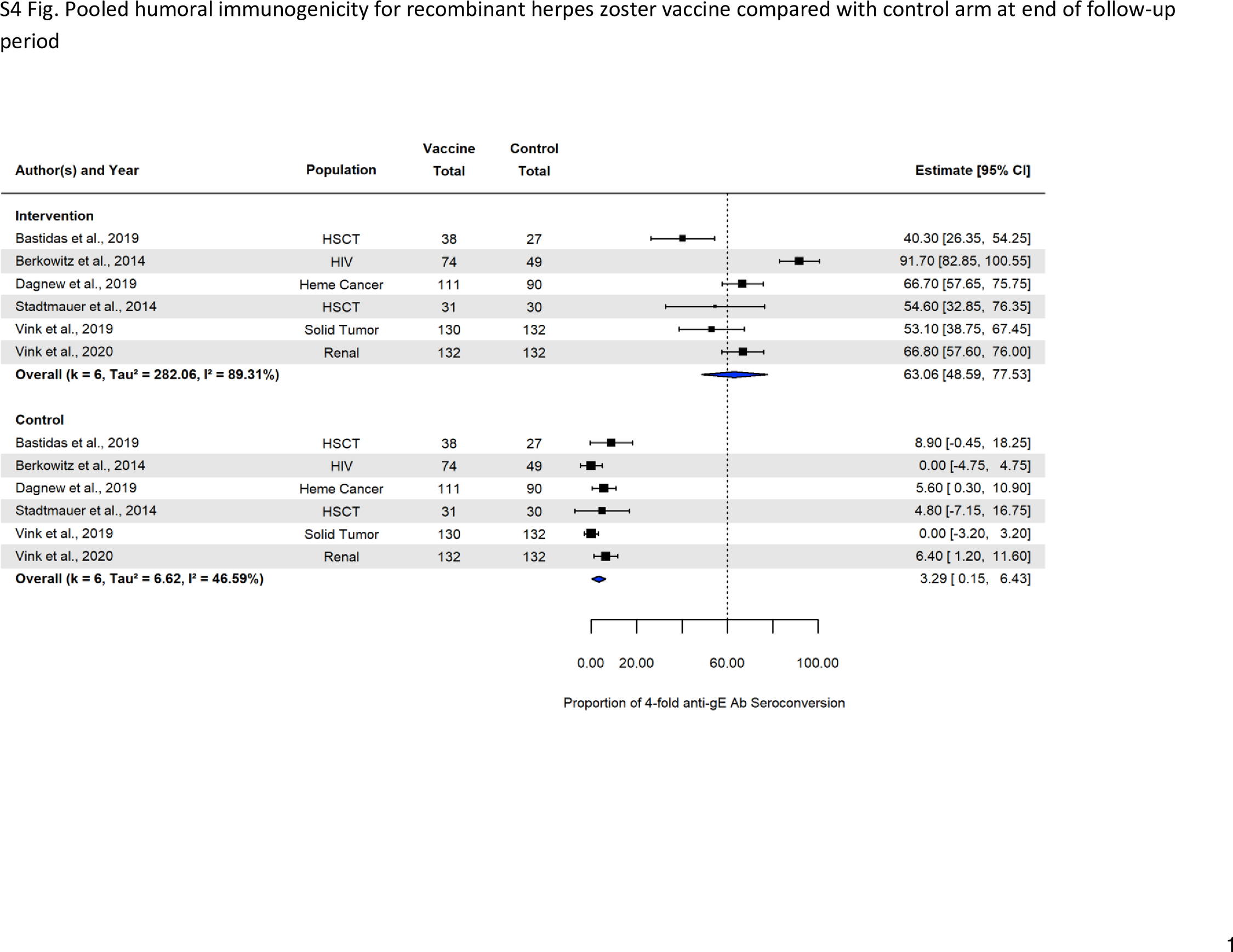

Supplement: S4 Fig — Long-term data on humoral immunogenicity with the vaccine and control arms. (TIF) [file pone.0313889.s004.tif]

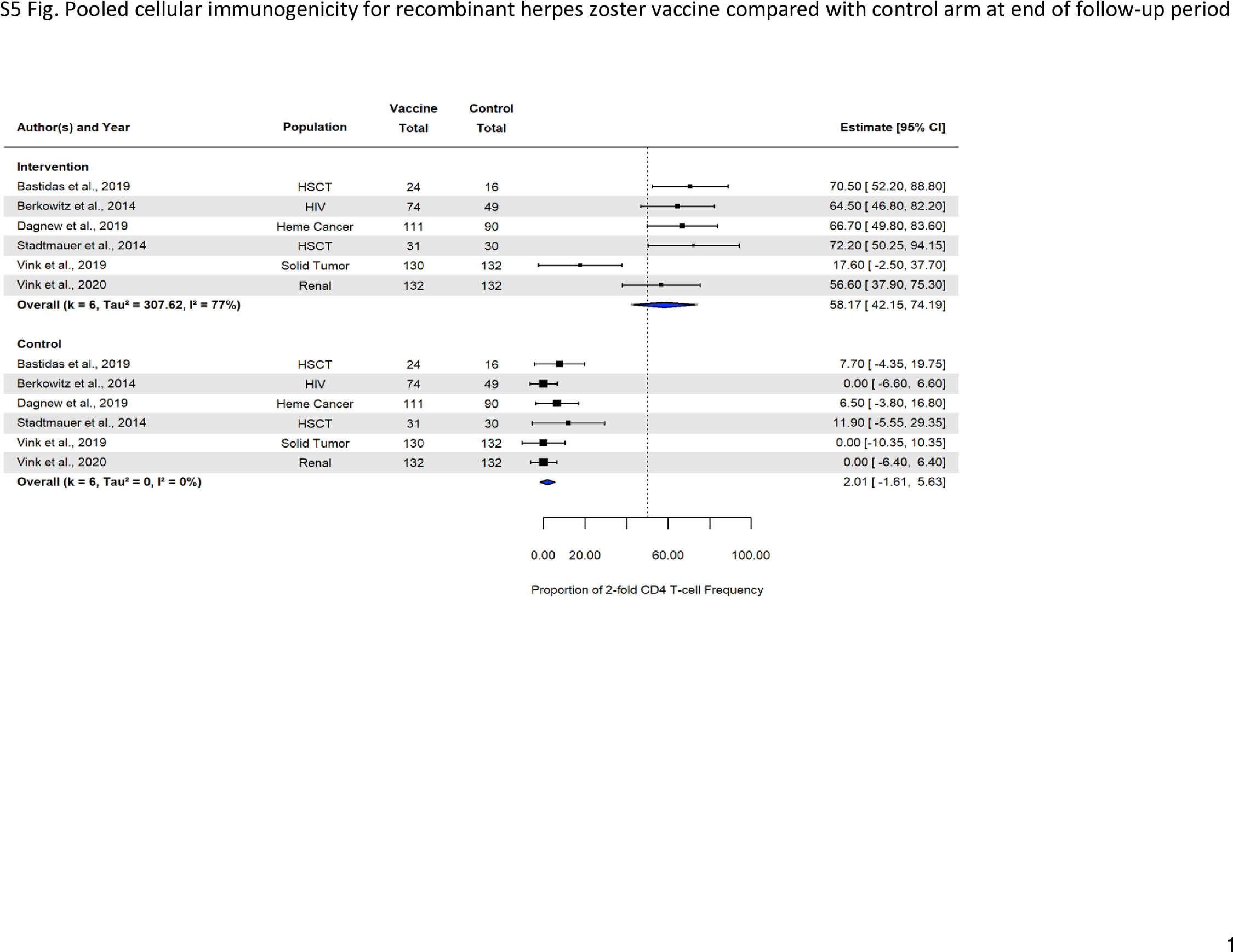

Supplement: S5 Fig — Long-term data on cellular immunogenicity with the vaccine and control arms. (TIF) [file pone.0313889.s005.tif]

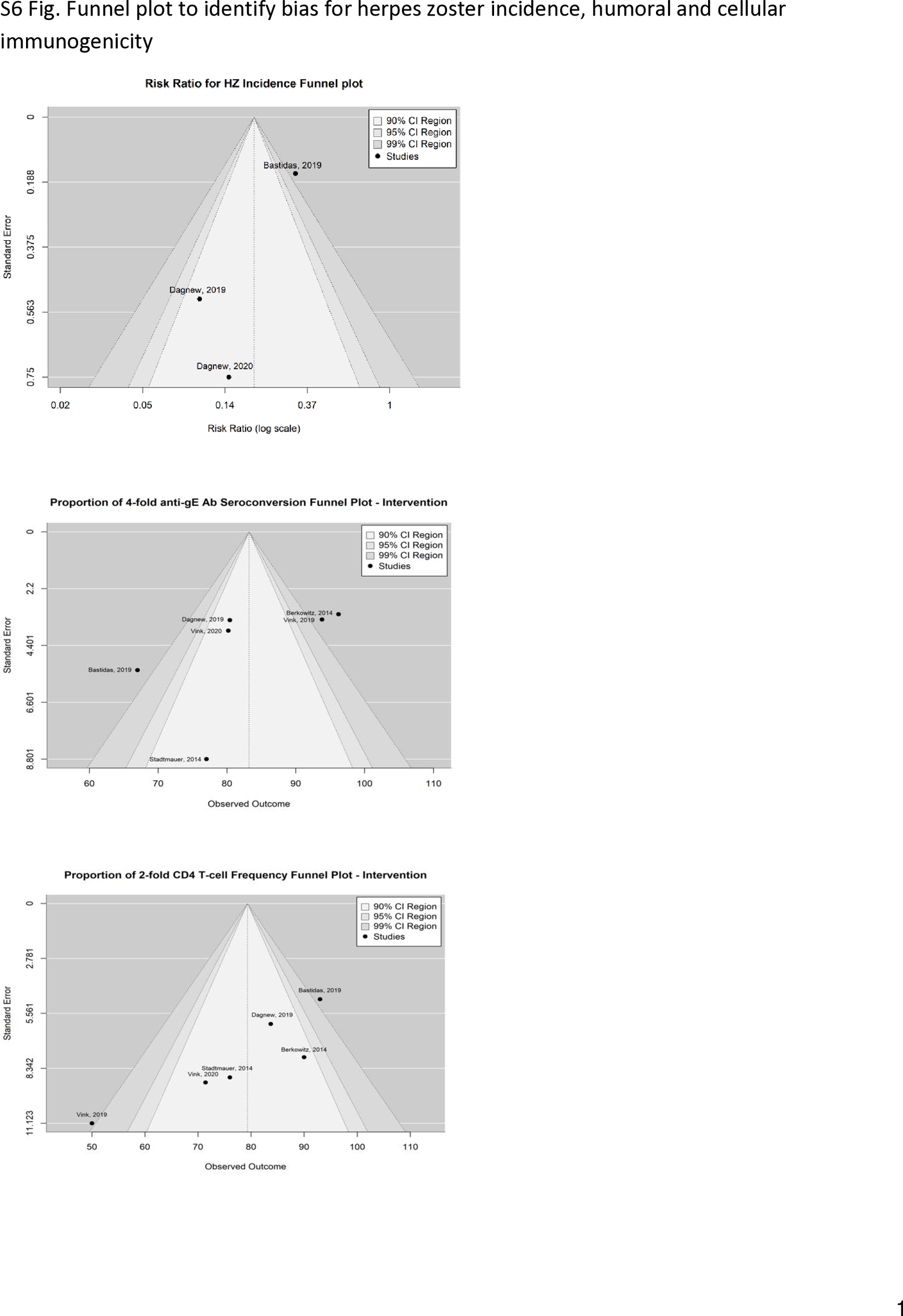

Supplement: S6 Fig — Visualization of bias associated with the included studies. (TIF) [file pone.0313889.s006.tif]
