# Supplementary material for: Systematic review and meta-analysis of recombinant herpes zoster vaccine in immunocompromised populations
Source: PLoS One. 2024 Nov 25;19(11):e0313889. doi: 10.1371/journal.pone.0313889 (PMC11588208; doi:10.1371/journal.pone.0313889)
Supplement: S1 Table — All studies identified in the literature search. (DOCX) [file pone.0313889.s007.docx]

S1 Table. Search strategies

Time period: 1984 to present

Databases: Embase, Medline, PubMed, Scopus, Web of Science, CINAHL, Cochrane CENTRAL

PICO Question:

P: Immunocompromised patients (including organ/tissue transplant patients, radiotherapy patients, immunosuppressive therapy)

I: Recombinant herpes zoster vaccine or Shingrix or recombinant zoster or recombinant shingles

C: Placebo or Live zoster vaccine or Inactivated zoster vaccine

O: Herpes zoster infection or shingles infection or adverse reactions

**Embase – Search terms:**

| **#** | **Query** | **Results** |
| --- | --- | --- |
| 1 | exp immune deficiency/ or exp immunosuppressive treatment/ or exp immunocompromised patient/ | 573,787 |
| 2 | (immunocompromis* or immunocompromiz* or immunosuppress* or immunodeficien* or immuno depress* or immunodefect* or immunodeficit* or immunoincompeten*).mp. | 1,033,139 |
| 3 | (immun* adj3 (weak* or compromis* or compromiz* or suppress* or deficien* or defect* or deficit* or depress* or incompeten* or disease* or disorder* or dysfunction*)).mp. | 439,527 |
| 4 | exp autoimmune disease/ or exp organ transplantation/ or exp tissue transplantation/ or exp xenograft/ or rheumatology/ or exp steroid/ or exp radiotherapy/ or exp chemotherapy/ | 4,394,312 |
| 5 | (autoimmun* or auto immun* or transplant* or rheumatol* or steroid* or chemotherap* or radiation or radiotherap* or HIV or AIDS).mp. | 4,555,961 |
| 6 | exp *herpes zoster/ and recombinant vaccine/ | 58 |
| 7 | ((recombinant adj3 shingles) or (recombinant adj3 zoster) or (recombinant adj3 shingrix) or (shingrix adj3 vaccine) or (recombinant adj3 HZ*) or (adjuvant adj3 shingrix) or ((herpes or zoster or shingles) adj5 (recombinant or shingrix))).mp. | 1,044 |
| 8 | 1 or 2 or 3 or 4 or 5 | 6,737,521 |
| 9 | 6 or 7 | 1,063 |
| 10 | 8 and 9 | 359 |
| 11 | limit 10 to (human and (embase or "preprints (unpublished, non-peer reviewed)")) | 189 |

**MEDLINE – Search terms:**

| **#** | **Query** | **Results** |
| --- | --- | --- |
| 1 | exp Immunocompromised Host/ or exp Immunologic Deficiency Syndromes/ or exp Immunosuppression Therapy/ | 457,418 |
| 2 | (immunocompromis* or immunocompromiz* or immunosuppress* or immunodeficien* or immuno depress* or immunodefect* or immunodeficit* or immunoincompeten*).mp. | 529,344 |
| 3 | (immun* adj3 (weak* or compromis* or compromiz* or suppress* or deficien* or defect* or deficit* or depress* or incompeten* or disease* or disorder* or dysfunction*)).mp. | 193,848 |
| 4 | exp Autoimmune Diseases/ or exp organ transplantation/ or exp tissue transplantation/ or exp transplantation, heterologous/ or exp Rheumatology/ or exp Rheumatic Diseases/ or exp Steroids/ or exp Radiotherapy/ or exp Chemotherapy/ | 3,429,409 |
| 5 | (autoimmun* or auto immun* or transplant* or rheumatol* or steroid* or chemotherap* or radiation or radiotherap* or HIV or AIDS).mp. | 3,226,577 |
| 6 | exp Herpes Zoster/ and exp recombinant proteins/ | 140 |
| 7 | ((recombinant adj3 shingles) or (recombinant adj3 zoster) or (recombinant adj3 shingrix) or (shingrix adj3 vaccine) or (recombinant adj3 HZ*) or (adjuvant adj3 shingrix) or ((herpes or zoster or shingles) adj5 (recombinant or shingrix))).mp. | 789 |
| 8 | 1 or 2 or 3 or 4 or 5 | 5,685,790 |
| 9 | 6 or 7 | 836 |
| 10 | 8 and 9 | 234 |
| 11 | limit 10 to humans | 165 |

**PubMed – Search terms:**

| **#** | **Query** | **Results** |
| --- | --- | --- |
| 1 | "immunocompromised host"[MeSH Terms] OR "immunosuppression therapy"[MeSH Terms] OR hosts, immunocompromised[MeSH Terms] OR host, immunocompromised[MeSH Terms] OR immunocompromised hosts[MeSH Terms] OR immunocompromised patient[MeSH Terms] OR immunosuppressed host[MeSH Terms] OR host, immunosuppressed[MeSH Terms] OR immunosuppressants[MeSH Terms] | 190,084 |
| 2 | immunocompromis* OR immunocompromiz* or immunodeficien* OR immunosuppress* OR immunoincompeten* or immunodepress* or immunodeficit* or immunodefect* | 777,994 |
| 3 | "immune deficiency"[Title/Abstract:~3] OR "immune deficit"[Title/Abstract:~3] OR "immune compromise"[Title/Abstract:~3] OR "immune suppressed"[Title/Abstract:~3] OR "immune suppression"[Title/Abstract:~3] OR "immune depressed"[Title/Abstract:~3] OR "immune depression"[Title/Abstract:~3] OR "immune deficient"[Title/Abstract:~3] | 36,330 |
| 4 | "transplant recipients"[MeSH Terms] OR "transplants"[MeSH Terms] OR "organ transplantation"[MeSH Terms] OR "transplantation, heterologous"[MeSH Terms] OR "acquired immunodeficiency syndrome"[MeSH Terms] OR "autoimmune diseases"[MeSH Terms] OR "autoimmune diseases"[MeSH Terms] OR "rheumatology"[MeSH Terms] OR "rheumatic diseases"[MeSH Terms] OR "steroids"[MeSH Terms] OR "radiotherapy"[MeSH Terms] | 2,116,942 |
| 5 | "autoimmun*"[All Fields] OR ("auto"[All Fields] AND "immun*"[All Fields]) OR "transplant*"[All Fields] OR "rheumatol*"[All Fields] OR "steroid*"[All Fields] OR "chemotherap*"[All Fields] OR "radiation"[All Fields] OR "radiations"[All Fields] OR "radiotherap*"[All Fields] OR "hiv"[All Fields] OR "acquired immunodeficiency syndrome"[All Fields] OR "aids"[All Fields] | 3,689,049 |
| 6 | shingles vaccine[MeSH Terms] AND recombinant vaccines[MeSH Terms] | 114 |
| 7 | shingrix OR recombinant zoster vaccine OR recombinant shingles vaccine OR recombinant herpes zoster vaccine OR herpes zoster recombinant vaccine OR zoster recombinant vaccine OR shingles recombinant vaccine OR shingrix recombinant vaccine OR recombinant zoster OR recombinant shingles OR recombinant herpes zoster or recombinant shingrix | 1,051 |
| 8 | "recombinant shingles"[Title/Abstract:~3] OR "recombinant zoster"[Title/Abstract:~3] OR "recombinant shingrix"[Title/Abstract:~3] OR "shingrix vaccine"[Title/Abstract:~3] OR "shingrix vaccination"[Title/Abstract:~3] OR"recombinant HZV"[Title/Abstract:~3] OR "adjuvant shingrix"[Title/Abstract:~3] | 317 |
| 9 | 1 OR 2 OR 3 OR 4 OR 5 | 5,060,091 |
| 10 | 6 OR 7 OR 8 | 1,051 |
| 11 | 9 AND 10 | 282 |
| 12 | 11 Filters: Humans | 235 |

**Web of Science – Search terms**

| **Web of Science Core Collection - Editions:**  Science Citation Index Expanded (WOS.SCI) – 1900 to 2023  Conference Proceedings Citation Index (WOS.ESCI) – 2018 to 2023  Emerging Sources Citation Index (WOS.ISTP) – 1990 to 2023 | | |
| --- | --- | --- |
| **#** | **Query** | **Results** |
| 1 | TS=(immunocompromis* or immunocompromiz* or immunosuppress* or immunodeficien* or immunodepress* or immunodefect* or immunodeficit* or immunoincompeten* or autoimmun* or auto immun* or transplant* or xenograft or rheumatol* or steroid* or chemotherap* or radiation or radiotherap* or HIV or AIDS) | 4,191,401 |
| 2 | TS=(immun* NEAR/3 (weak* or compromis* or compromiz* or suppress* or deficien* or defect* or deficit* or depress* or incompeten* or disease* or disorder* or dysfunction*)) | 192,873 |
| 3 | TS=((recombinant NEAR/3 shingles) or (recombinant NEAR/3 zoster) or (recombinant NEAR/3 herpes zoster) or (recombinant NEAR/3 shingrix) or (shingrix NEAR/3 vaccine) or (recombinant NEAR/3 HZV*) or (adjuvant NEAR/3 shingrix) or (shingrix NEAR/3 vaccination)) | 343 |
| 4 | DT=(Article) | 46,589,512 |
| 5 | 1 or 2 | 4,291,505 |
| 6 | 3 and 4 and 5 | 70 |

**Scopus – Search terms**

| **#** | **Query** | **Results** |
| --- | --- | --- |
| 1 | TITLE-ABS-KEY=(immunocompromis* or immunocompromiz* or immunosuppress* or immunodeficien* or immunodepress* or immunodefect* or immunodeficit* or immunoincompeten* or autoimmun* or auto immun* or transplant* or xenograft or rheumatol* or steroid* or chemotherap* or radiation or radiotherap* or HIV or AIDS) | 1,157,914 |
| 2 | TITLE-ABS-KEY=(immun* W/3 (weak* or compromis* or compromiz* or suppress* or deficien* or defect* or deficit* or depress* or incompeten* or disease* or disorder* or dysfunction*)) | 434,355 |
| 3 | TITLE-ABS-KEY=((recombinant W/3 shingles) or (recombinant NEAR/3 zoster) or (recombinant W/3 herpes zoster) or (recombinant W/3 shingrix) or (shingrix W/3 vaccine) or (recombinant W/3 HZV*) or (adjuvant W/3 shingrix) or (shingrix W/3 vaccination)) | 343 |
| 4 | 1 or 2 | 1,346,122 |
| 5 | 3 and 4 and 5 | 122 |

**CINAHL Complete – Search terms**

Search mode: Boolean/Phrase

| **#** | **Query** | **Results** |
| --- | --- | --- |
| 1 | (MH "Immunosuppressive Agents+") OR (MH "Immunosuppression+") OR (MH "Immunocompromised Host") OR (MH "Immunologic Deficiency Syndromes+") | 134,701 |
| 2 | immunocompromis* OR immunocompromiz* OR immunodeficien* OR immunodeficit* OR immunosuppress* OR immunodepress* OR immunoincompeten* OR immunodefect* | 80,964 |
| 3 | immun* N3 weak* OR immun* N3 dysfunction* OR immun* N3 disorder* OR immun* N3 disease* OR immun* N3 incompeten* OR immun* N3 depress* OR immun* N3 deficit* OR immun* N3 defect* OR immun* N3 deficien* OR immun* N3 suppress* OR immun* N3 compromiz* OR immun* N3 compromis* | 34,104 |
| 4 | MH "Radiotherapy+" OR MH "Rheumatic Diseases+" OR MH "Rheumatology" OR MH "Transplant Recipients" OR MH "Transplantation+" OR MH "Autoimmune Diseases+" | 271,740 |
| 5 | autoimmun* or auto immun* or transplant* or rheumatol* or steroid* or chemotherap* or radiation or radiotherap* or HIV or AIDS | 571,148 |
| 6 | (MH "Herpes Zoster Vaccine" OR MH "Herpes Zoster+") AND (MH "Recombinant Proteins+" OR MH "Glycoproteins+") | 65 |
| 7 | (recombinant N3 shingles) or (recombinant N3 zoster) or (recombinant N3 shingrix) or (shingrix N3 vaccine) or (recombinant N3 HZ*) or (adjuvant N3 shingrix) or ((herpes or zoster or shingles) N5 (recombinant or shingrix)) | 149 |
| 8 | 1 or 2 or 3 or 4 or 5 | 749,963 |
| 9 | 6 or 7 | 200 |
| 10 | 8 and 9 and Limiters - Human | 31 |

**Cochrane Central Register of Controlled Trials (CCTR) – Search terms:**

1991 - present

| **#** | **Query** | **Results** |
| --- | --- | --- |
| 1 | exp Immunocompromised Host/ or exp Immunologic Deficiency Syndromes/ or exp Immunosuppression Therapy/ | 19281 |
| 2 | (immunocompromis* or immunocompromiz* or immunosuppress* or immunodeficien* or immuno depress* or immunodefect* or immunodeficit* or immunoincompeten*).mp. | 33761 |
| 3 | (immun* adj3 (weak* or compromis* or compromiz* or suppress* or deficien* or defect* or deficit* or depress* or incompeten* or disease* or disorder* or dysfunction*)).mp. | 12509 |
| 4 | exp Autoimmune Diseases/ or exp organ transplantation/ or exp tissue transplantation/ or exp transplantation, heterologous/ or exp Rheumatology/ or exp Rheumatic Diseases/ or exp Steroids/ or exp Radiotherapy/ or exp Chemotherapy/ | 268591 |
| 5 | (autoimmun* or auto immun* or transplant* or rheumatol* or steroid* or chemotherap* or radiation or radiotherap* or HIV or AIDS).mp. | 245828 |
| 6 | exp Herpes Zoster/ and exp Vaccines, Synthetic/ | 23 |
| 7 | ((recombinant adj3 shingles) or (recombinant adj3 zoster) or (recombinant adj3 shingrix) or (shingrix adj3 vaccine) or (recombinant adj3 HZ*) or (adjuvant adj3 shingrix) or ((herpes or zoster or shingles) adj5 (recombinant or shingrix))).mp. | 120 |
| 8 | 1 or 2 or 3 or 4 or 5 | 452083 |
| 9 | 6 or 7 | 122 |
| 10 | 8 and 9 | 60 |
