# Supplementary material for: Systematic review and meta-analysis of recombinant herpes zoster vaccine in immunocompromised populations
Source: PLoS One. 2024 Nov 25;19(11):e0313889. doi: 10.1371/journal.pone.0313889 (PMC11588208; doi:10.1371/journal.pone.0313889)
Supplement: S2 Table — RZV–recombinant zoster vaccine; PLB–placebo; GM ratio–geometric mean ratio; anti-gE–anti glycoprotein E Antibody Concentration; IRR–incidence rate ratio; Pre-Chemo–Before chemotherapy. (DOCX) [file pone.0313889.s008.docx]

S2 Table. Results for secondary outcomes at end of follow up period

| Author, year | Number of events of PHN | Incidence of PHN | Cumulative follow-up (months) | Number of events of HZ | Incidence of HZ | Percentage of patients with a response rate to anti-gE humoral immune, % (95% CI) | Adjusted GM ratio for anti-gE Ab concentrations (95% CI) | Percentage of patients with a response rate to cell-mediated immunity, % (95% CI) | GM ratio for anti-gE CD4[2+] T cell concentration (95% CI) |
| --- | --- | --- | --- | --- | --- | --- | --- | --- | --- |
| Stadtmauer, 2014 | ---- | ---- | 15 mon | ---- | ---- | At month 15  1) 3 doses ge/AS01B:  66.7% (46.0 - 83.6)  2) 3 doses gE/AS01E:  77.3% (54.7 – 92.3)  3) 2 doses gE/AS01B:  54.6 % (32.3 – 75.8)  4) 3 doses placebo:  4.8% (0.0 - 23.9) | At month 15  1) 3 doses ge/AS01B: 28.70 (10.92-75.46)  2) 3 doses gE/AS01E:  21.98 (7.87-61.37)  3) 2 doses gE/AS01B:  8.81 (3.41-22.80) | At month 15  1) 3 doses ge/AS01B:  95.9% (79.6 - 100)  2) 3 doses gE/AS01E:  83.4% (58.7 - 96.6)  3) 2 doses gE/AS01B:  72.2% (46.6 - 90.5)  4) 3 doses placebo:  11.9% (1.6 -36.5) | At month 15  1) 3 doses ge/AS01B:  15.15 (8.33-27.54)  2) 3 doses gE/AS01E:  11.39 (6.68-19.42)  3) 2 doses gE/AS01B:  5.03 (2.74-9.26) |
| Bastidas, 2019 | At month 24  RZV: 1/870  PLB:  9/851 | At month 24  IRR: 0.11 (0.01-0.78) | 24 mon | At month 13  RZV: 30/682  PLB: 100/604 | At month 13  0.27 (0.18-0.39) | At month 13  RZV: 40.3% (27.0-54.9)  PLB: 8.9% (2.6-21.3)  At month 25  RZV: 44.9 (28.3-61.8)  PLB: 14.8 (4.2-33.3) | ---- | At month 13  RZV: 70.5% (49.8-86.4)  PLB: 7.7% (1.1-25.2)  At month 25  RZV: 70.8 (48.9-87.5)  PLB: 12.4 (1.6-38.2) | ---- |
| Dagnew, 2019 | ---- | ---- | 15 mon | At month 13  RZV: 2/286  PLB: 14/283 | At month 13  RZV: 8.5 per 1000-person years  PLB: 66.2 per 1000-person years  VE: 87.2 (44.3-98.6) | At month 13  RZV: 66.7% (57.1-75.2) or 74/111  PLB: 5.6% (1.9-12.5) or 5/90 | ---- | At month 13  RZV: 66.7% (48.2-82.0) or 22/33  PLB: 6.5% (0.8-21.4) or 2/31 | ---- |
| Dagnew, 2020 | ---- | ---- | 24 mon | At end of the study    RZV: 4/936  PLB: 38/923 |  | ---- | ---- | ---- | ---- |
| Vink, 2019 | ---- | ---- | 15 mon | ---- | ---- | At month 13  Pre-Chemo  RZV: 53.1% (38.5-67.2)  PLB: 0% (0-6.4)  Pre- & On-Chemo  RZV: 51.5% (39.0-63.8)  PLB: 0% (0-5.1) | ---- | At month 13  Pre-Chemo RZV: 17.6% (3.2-43.4) PLB: 0% (0-20.7)  Pre- & On-Chemo  RZV: N/A  PLB: N/A | ---- |
| Vink, 2020 | ---- | ---- | 15 mon | ---- | ---- | At month 13  RZV: 66.8 (57.0-75.4)  PLB: 6.4 (2.4-12.8) | ---- | At month 13  RZV: 56.6 (37.1-74.5)  PLB: 0 (0-12.8) | ---- |
| Berkovitz, 2014 | ---- | ---- | 18 mon | ---- | ---- | At month 18  RZV: 91.7 (80-97.7)  PLB: 0 (0-9.5) | ---- | At month 18  RZV: 64.5 (45.4-80.8)  PLB: 0 (0-13.2) | ---- |

Legend: RZV – recombinant zoster vaccine; PLB – placebo; GM ratio – geometric mean ratio; anti-gE – anti glycoprotein E Antibody Concentration; IRR – incidence rate ratio; Pre-Chemo – Before chemotherapy;
