# Supplementary material for: Systematic review and meta-analysis of recombinant herpes zoster vaccine in immunocompromised populations
Source: PLoS One. 2024 Nov 25;19(11):e0313889. doi: 10.1371/journal.pone.0313889 (PMC11588208; doi:10.1371/journal.pone.0313889)
Supplement: S3 Table — Details of the seven studies included in this review. (DOCX) [file pone.0313889.s009.docx]

# S3 Table. Narrative of the included studies

| ***Hematopoietic stem cell transplant (HSCT)*** | |
| --- | --- |
| Stadtmauer, 2014 | Stadtmauer et al. conducted the first of the studies, a phase 1/2 study of RZV in 121 autologous hematopoietic stem cell transplant recipients (HSCT).^37^ In this randomized, single-blinded, placebo-controlled study, adults 18 years of age or older who had undergone an HSCT within the last 50 to 70 days, and had no previous history of HZ or known exposure to VZV since transplantation were randomized to receive 2 doses (months 0, 3) or 3 doses (months 0, 1, 3) of RZV. Humoral and cellular immune responses were evaluated at months 1, 2, 3, and 15 post-vaccination. The study showed that gE-titers were immunogenic even if given soon after HCT, and immune responses persisted for up to 1 year, suggesting sustained HZ protection. The levels of anti-gE GMCs and frequencies of CD4[2+] T cells were comparable with those in immunocompetent adults ≥50 years of age immunized with 2 doses of RZV. gE-specific humoral and cellular immune responses were higher in the 3-dose group than in the 2-dose group at all time points; however, the additional increase after the third dose was modest, prompting a 2-dose vaccine schedule for subsequent clinical trials.  Within 30 days of vaccination, 75.9% to 83.3% of RZV subjects and 51.6% to 70.0% of placebo subjects reported unsolicited adverse events, the most common one being chills. Serious side effects included recurrence of malignancy in 32 subjects (13 in the 3-dose RZV group, 11 in the 2-dose RZV group, and 8 in the placebo group). Immune-mediated diseases were not seen in the study. |
| Bastidas, 2019 | Bastidas et al. used data from the phase 1/2 study to design a phase 3 randomized, single-blind, placebo-controlled, multicenter study to evaluate RZV vaccine immunogenicity, efficacy and safety in HSCT recipients.^38^ Individuals aged 18 years and older who had undergone autologous HSCT in the previous 50 to 70 days were eligible to receive 2 doses of RZV. The investigators evaluated humoral and cellular immune responses. Efficacy was defined as the incidence rate ratio (IRR) for the development of HZ or complications (PHN, other HZ–related complications, and hospitalizations). Solicited and unsolicited adverse events were evaluated for all participants who received at least one dose of the study vaccine or placebo.  A total of 1846 participants received at least one dose, but the primary analysis was a modified intention to treat analysis, which was the cohort that received both vaccine doses (N=1721). Over a median follow-up of 21 months, starting 1 month after dose 2, a total of 135 confirmed cases of HZ occurred in the placebo arm versus 49 cases in the RZV arm (IRR 0.32; 95% CI: 0.22-0.44), translating to a vaccine efficacy of 68.2% (95% CI: 55.6-77.5). The vaccine efficacy for prevention of PHN was 89% (95% CI: 22-100) and 85% (95% CI: 32–97) for prevention of HZ-related hospitalization. Strong and sustained humoral and cellular immune responses were seen in the RZV arm compared to the placebo group, consistent with previous observations. One month after dose 2, all RZV recipients had detectable gE–specific antibodies and CD4[2+] T-cells that remained higher than baseline 24 months after the second dose.  Like the original clinical trials in immunocompetent patients, solicited injection site reactions were seen in 85% of RZV recipients compared to 10% of placebo recipients, while systemic symptoms (30-days postvaccination) were 75% and 51% after RZV and placebo, respectively. Reactions were transient and lasted 2 to 3 days. Graft-versus-host disease was not seen in either arm, immune-mediated diseases occurred in 1.4% and 0.9% of patients, relapse of malignancy in 15.7% and 16.1% of patients in the RZV and placebo arm, respectively. The investigators concluded that RZV was efficacious and safe to use in the severely immunocompromised population, and that the 2-dose series would allow for greater compliance with vaccine uptake. |
| ***Hematological malignancies*** | |
| Dagnew, 2019 | Dagnew and colleagues studied RZV vaccine efficacy and safety in individuals with hematological malignancies through another randomized, observer-blinded, placebo-controlled clinical trial.^39^ Participants 18 years of age or older were randomized to receive 2 doses of the RZV (0, and 1 or 2 months later) or placebo during or after chemotherapy. The outcomes were herpes zoster incidence and immunogenicity in all participants except those with non-Hodgkin B-cell lymphoma and chronic lymphocytic leukemia.  Two cases of HZ were seen in the RZV arm and 14 cases in the placebo arm. A post-hoc analysis, based on all study participants, including patients with non-Hodgkin B-cell lymphoma and chronic lymphocytic leukemia, revealing an HZ incidence of 8·5 per 1000 person-years in the vaccine group and 66·2 per 1000 person-years in the placebo group, resulting in a vaccine efficacy of 87·2% (95% CI 44·3–98·6).  In their cohort, 119 of the 148 (80·4%) participants had a humoral vaccine response to RZV compared with 1 of 130 (0·8%) participants in the placebo group at 30 days post-second dose of vaccination. Adjusted GM’s of anti-glycoprotein E antibodies was much higher at 23,132·9 mIU/mL (95% CI 16,642·8–32,153·9) in the vaccine group and 777·6 mIU/mL (702·8–860·3) in the placebo group (adjusted geometric mean ratio 29·75, 21·09–41·96; p<0·0001). Similar data were seen for cellular immunity at month 2. The median CD4[2+] T-cell frequencies increased from 77.5 (IQR 1·0–191·4) to 3081·9 (IQR 1766·2–7413·6) in the vaccine group, but did not change for the placebo arm with the pre-vaccination median CD4[2+] T-cell frequency of 101·2 (IQR 1·0–193·1) and post-vaccination median of 99·1 (IQR 1·0–268·3). Immune responses persisted above baseline for RZV until month 13 in all strata.  The vaccine was more reactogenic than the placebo at 7 and 30 days after vaccination. Pain was reported by 221 [79·5%] of 278 vaccine group participants and 45 [16·4%] of 274 placebo group participants; fatigue was reported by 162 [58·3%] of 278 vaccine group participants and 102 [37·2%] of 274 placebo group participants). Incidences of unsolicited and serious adverse events were similar between the groups. The following Immune-mediated diseases were reported in 3/283 (1.1%) participants in the vaccine arm: autoimmune pancytopenia, gout, and erythema nodosum, while autoimmune hemolytic anemia and Guillain-Barré syndrome were reported in 2/279 (0.7%) placebo arm. From the first vaccination until study end, relapse or progression of the original hematological malignancy was reported in 45 (15·9%) participants in the vaccine group and 58 (20·8%) participants in the placebo group. |
| ***Individuals with immune-mediated diseases*** | |
| Dagnew, 2020 | Dagnew et al.^40^ conducted a posthoc analysis using a smaller number of patients from the original ZOE clinical trials^24,25^ who they considered to fall under the category of participants who had at least one immune-mediated disease at the time of enrolment. The most frequent pre-existing conditions were psoriasis, spondyloarthropathy, rheumatoid arthritis and celiac disease; their prevalence was balanced between study groups. From the two ZOE studies, 983/14,645 in the RZV arm and 960/14,660 in the placebo arm reported at least one immune-mediated disease. In this group of participants, vaccine efficacy for the two-dose recipients against HZ was 90.5% (95% CI: 73.5-97.5). Immunogenicity data were not available.  Local or systemic adverse events were not reported for the post-hoc analysis, only serious events were reported. The proportion of serious adverse events were similar in the vaccine (14.6%) and placebo (11.7%) arms and included pneumonia, myocardial infarction, urinary tract infection, and cardiac failure. In the overall ZOE-50/70 population, infections and cardiac disorders were also the two most common side effects.^44^ Similar to the overall pooled ZOE-50/ZOE-70 studies, fatal serious adverse events were reported for 5.1% (95% CI: 3.8-6.7) of the RZV and 6.6% (95% CI: 5.1-8.3) of the placebo recipients during the entire study period.^44^ Finally, possible flare-ups of the preexisting immune condition remained the same in the vaccine and placebo arms at 27 patients (2.8%) for each group. New onset of a different immune condition was reported in 16 (1.6%) of RZV patients compared to 23 (2.4%) of the placebo patients. The authors of this study concluded that RZV was highly efficacious and safe in individuals with preexisting immune disorders. |
| ***Solid tumors and Transplant*** | |
| Vink, 2019  Vink, 2020 | *Solid tumors*  Vick et al^41^ conducted a similarly designed study to Dagnew and colleagues^39^ but evaluated individuals with solid tumors. In their randomized, observer-blinded, placebo-controlled clinical trial, participants 18 years of age or older were eligible for participation if they had been diagnosed with 1 or more solid organ tumors, and were receiving or scheduled to receive cytotoxic or immunosuppressive chemotherapy. Participants were excluded from the study if they were scheduled to receive targeted therapies without immunosuppressive medications, were on corticosteroids, had previous chemotherapy in the past 28 days, had a history of HZ or VZV, received zoster-related vaccination (shingles or chickenpox) in the 12 months before enrollment. Recombinant zoster vaccine was studied as a 2-dose regimen (0, and 1 or 2 months later) or placebo that was evaluated for immunogenicity and safety at month 2 (i.e., 30 days after the second dose). The primary outcome was humoral immunity, with cellular immunity and safety as secondary outcomes in pre-chemotherapy participants, defined as individuals who received the first vaccine dose 8 to 30 days before the start of a chemotherapy cycle. A total of 262 participants were randomized in this study. Approximately 45% of the participants were diagnosed with breast cancer, and 20% had colorectal cancer; other cancers included lung, prostate, bladder, pancreas and others.  One hundred and eighty-five participants were evaluated for humoral immunogenicity. At month 2, the adjusted GM ratio (RZV over placebo) was significantly higher for the vaccine recipients (adjusted GM ratio 23.3 [95% CI, 17.9-30.0]) in the pre-chemo group. The GM ratio remained high at 14.4 (95% CI, 10.7-19.5) for RZV recipients when evaluating the two subgroups together (pre-chemo and on-chemo), but it should be noted that GMs were higher in the RZV pre-chemo group than in the RZV-OnChemo group. The humoral VRR to RZV was 93.8% (95% CI, 85.0%-98.3%) at month 2 in the RZV pre-chemo group. No increases in GMs were observed in placebo recipients at any time point. Results for cellular immunity were the same as humoral with frequencies of gE-specific CD4[2+] T cells per 10^6^ and total CD4+ T cells being higher in the vaccine arm than placebo arm at month 2 and 13; once again, levels in the pre-chemo group were higher than the on-chemo group. At month 2, the CMI VRR was 50.0% (95% CI, 28.2%-71.8%) in RZV recipients.  Solicited adverse events, both local and systemic, were more frequent among RZV recipients than placebo recipients. Incidence of unsolicited adverse events (RZV: 85.5%, PLB: 89.6%), serious adverse events (RZV: 13.7%, PLB: 12.2%), deaths (RZV: 10.3%, PLB: 9.6%), and potential immune-mediated diseases (RZV: 0%, PLB: 0.9%), were similar between RZV and placebo recipients. The authors concluded that despite the presence of cancer and/or chemotherapy that could potentially reduce vaccine immune response, RZV induces both humoral and cellular immunity. Administration of the first RZV dose 8-30 days before chemotherapy elicits a stronger response than administration while on chemotherapy.  *Solid organ transplants (SOT)*  Efficacy of RZV in solid organ transplant (SOT) individuals was studied by Vick et al.^42^ in a randomized, observer-blinded, placebo-controlled clinical trial. Participants 18 years of age or older were eligible for participation if they had received a renal transplant in the 4-18 months prior, ABO-compatible allograft, had stable renal function, and had not experienced allograft rejection in the past 3 months. Participants were excluded from the study if they had primary kidney disease, which is known to have a high incidence of recurrence, multiple organs transplanted, autoimmune or immune-mediated disease, history of HZ or VZV in the past, received HZ/VZV vaccination in the 12 months preceding enrollment.  Participants received 2 doses, 0.5mL each, of RZV vaccine or placebo that were given 1 to 2 months apart. Primary outcomes were immunogenicity and safety at month 2 (i.e., 30 days after the second dose); however, participants were followed till 12 months after the second dose. Humoral and cell-mediated immunity were assessed at all time points. Safety and reactogenicity were evaluated as solicited adverse events within the first 7 days post-vaccination, and unsolicited 30-day post-vaccination adverse events. Local and systemic adverse events were graded as mild (grade 1), moderate (grade 2) and severe (grade 3). Biopsy confirmed allograft rejections and immune-mediated disease were recorded as serious adverse events.  At randomization, the two groups were stratified according to vaccine/placebo receipt pre-chemotherapy or on-chemotherapy. The primary outcomes were humoral and cellular immunity and safety. One hundred and thirty-two participants were randomized into the RZV arm and 132 into the placebo arm. From a total of 264, 260 participated through to the last visit; demographic characteristics were balanced between the two study arms.  One month after the second dose the humoral vaccine response rate (VRR) in the RZV group was 80.2% (95% CI: 71.9%–86.9%), the adjusted anti-gE antibody GMC ratio (RZV over placebo) was 14.00 (95% CI: 10.90–17.99). Among RZV recipients, anti-gE antibody GMCs increased from 1354.4 mIU/mL at baseline to 19,163.8 mIU/mL at 1-month after the second dose, and persisted through to month 13 (or 12 months after second dose) at 8545.1 mIU/mL (95% CI: 6753.7–10 811.5 mIU/mL). Cell-mediated immunity was measured in a smaller sample of participants, but the results were similar to the humoral immune response at months 2 and 13. The vaccine response rate was 71.4% (95% CI, 51.3%–86.8%) in the RZV group at month 2, the geometric mean ratio (RZV over placebo) of gE-specific CD4[2+] T-cell frequencies was 17.26 (95% CI, 5.92–50.36) and the median CD4[2+] T-cell frequencies increased from 21.2 to 2149.0 and remained above baseline by month 13.  During the 7-day post-vaccination period, local injection events occurred in 87% of the RZV participants and 8.3% of the placebo participants; these lasted a median of 4 days. Systemic adverse events also occurred at higher frequencies in the RZV participants compared to the placebo arm (myalgia: 49.6% vs 23.5%; fever: 16% vs 3.8%). Unsolicited adverse events in the 30-days post-vaccination occurred in similar frequency between the two arms. 38.5% of RZV participants compared to 33.3% in the placebo group, with grade 3 events being reported by 5.3% of RZV recipients and 3.8% of placebo recipients. Serious adverse events occurred more commonly in the placebo arm (25%) than RZV (19.7%); no biopsy-proven rejections or immune-mediated diseases occurred from the first vaccination to 30 days after the last dose in either of the two groups. The authors concluded that RZV was immunogenic in renal transplant patients who were chronically immunosuppressed with medications. The safety data was in line with the reactogenicity profile seen in the RZV pre-licensure clinical trials,^24,25^ with mild to moderate local and systemic adverse events occurring more frequently in the vaccine arm. |
| ***Human immunodeficiency virus (HIV) positive individuals*** | |
| Berkovitz, 2014 | Berkowitz et al. conducted a phase 1/2 randomized, placebo-controlled study to evaluate the immunogenicity and safety of RZV in HIV-infected individuals aged ≥18 years.^43^ Randomization was stratified according to ART therapy and CD4+ T-cell counts: on stable ART therapy for one year with ≥200 cells/mm^3^ (N=94), on stable ART therapy for one year with 50–199 cells/mm^3^ (N=14), and ART-naive adults with a CD4+ T-cell count of ≥500 cells/mm^3^ (N=15). The outcomes were evaluated 30 days after the third dose (i.e., month 7) and the end of study (month 18). Subjects received 3 doses of RZV or placebo at months 0, 2, and 6.  In the overall study population, the anti-gE GMCs increased significantly in the RZV arm at month 7 (RZV: 63,812 mIU/mL (95% CI: 51,1183-79,557) vs PLB: 1028 mIU/mL (95% CI: 658-1605)). One year after the third dose, the anti-gE GMCs remained above the prevaccination concentrations in the RZV group. The GMC ratio at 7 months was 46.22 (90% CI: 33.63–63.53) for the vaccine group and ≤3 for the placebo group. In the RZV group, the vaccine response rate (proportions of subjects with anti-gE humoral vaccine responses) was between 92.3% and 98.1% at the different time points, whereas they were ≤2.8% in the placebo group; at month 7, the VRR was 96.2% and 2.8% in the RZV and placebo groups, respectively. Of note, the anti-gE GMCs at month 3 (i.e., after the second dose) were not significantly different from month 7 (i.e., after the third dose) with titers in the RZV arm of 50,443 mIU/mL (95% CI: 40,899-62,213) and a VRR of 98.1% (95% CI: 89.9-100).  Cell-mediated immune response was similar to the humoral immune response, with the median frequency of CD4[2+] T-cell counts increasing in the RZV group but not the placebo group at month 7 (RZV: 2578, PLB: 122). The GMC ratio for CD4[2+] T cell count at 7 months was 21.95 (70% CI: 12.97-38.02) for the vaccine group and ≤2 for the placebo group. In the RZV group, the vaccine response rate was 90% and 64% at months 7 and 18, respectively, whereas it was 16.7% and 0% in the placebo group at those time points. Two doses of RZV gave a median CD4+ T cell immune response of 2809 with a VRR of 85.7% (95% CI: 67.3-96.0).  Similar to the previous studies in immunocompromised and non-immunocompromised patients, local and systemic reactions were typical among RZV recipients. Pain at the injection site was experienced by 98.6% and 12.5% of the RZV and placebo groups, respectively, followed by fatigue (RZV: 75.3% PLB: 29.2%). However, these reactions were transient, and no subject withdrew from the study because of a solicited reaction. Over the study period, serious adverse events were reported in 8.1% of the vaccine group and 4.1% of the placebo group; none of them were considered by the investigator to be related to vaccination. Fourteen patients had worsening of their HIV disease at month 7, with 9 (12.2%) patients in the RZV arm and 5 (10.2%) in the placebo arm. These individuals had increased HIV RNA viral loads and/or decreased CD4 T-cell counts. However, these effects were transient and there were no sustained impacts on HIV disease control in the study groups.  The authors concluded that RZV elicits strong gE-specific cell-mediated and humoral immune responses in HIV-infected individuals after 3 doses and that the responses persisted over prevaccination levels at least 1 year after the last dose. Due to the small number of patients in each of their CD4 count specific-cohorts, the investigators were not able to give results according to the CD4 counts of less than 200/mm3 or above 200/mm3. They also commented that both the cellular and humoral immune responses did not increase significantly between the second and third doses, and therefore a 2-dose schedule may be a reasonable alternative in HIV-infected patients. The authors felt RZV had a comparable safety profile in HIV-infected patients to non-HIV patients with no sustained impact on viral load or CD4 counts. |
