# Supplementary material for: Systematic review and meta-analysis of recombinant herpes zoster vaccine in immunocompromised populations
Source: PLoS One. 2024 Nov 25;19(11):e0313889. doi: 10.1371/journal.pone.0313889 (PMC11588208; doi:10.1371/journal.pone.0313889)
Supplement: S4 Table — RZV–recombinant zoster vaccine; PLB–placebo; ADRs–adverse drug reactions; RR–risk ratio. (DOCX) [file pone.0313889.s010.docx]

# S4 Table. Results for adverse drug reactions

| Author, year | Local injection ADRs (solicited) within 7 days post-vaccination | Grade 3 Local injection ADRs (solicited) within 7 days post-vaccination | Systemic ADRs within 7 days post-vaccination | Grade 3 systemic ADRs within 7 days post-vaccination | Unsolicited ADRs within 30 days post-vaccination | Grade 3 unsolicited ADRs within 30 days post-vaccination | Serious adverse events from first visit to study end | Fatal ADRs from first visit to study end |
| --- | --- | --- | --- | --- | --- | --- | --- | --- |
| Stadtmauer, 2014 | ---- | ---- | ---- | ---- | ---- | ---- | RZV (2 dose): 10/28  PLB: 8/30 | ---- |
| Bastidas, 2019 | RZV: 773/901  PLB: 93/892  RR: 8.23 (6.78-9.99) | RZV: 128/901  PLB: 3/892  RR: 42.24 (13.50-132.21) | RZV: 687/901  PLB: 455/892  RR: 1.49 (1.39-1.61) | RZV: 119/901  PLB: 54/892  RR: 2.18 (1.60-2.97) | RZV: 360/922  PLB: 353/924  RR: 1.02 (0.91-1.15) | RZV: 60/922  PLB: 47/924  RR: 1.28 (0.88-1.85) | RZV: 68/922  PLB: 66/924 | 77/922  79/924 |
| Dagnew, 2019 | RZV: 233/278  PLB: 48/274  RR: 4.78 (3.68-6.22) | RZV: 37/278  PLB: 0/274  RR: 73.92 (4.56-1197.82) | RZV: 206/278  PLB: 134/274  RR: 1.52 (1.32-1.74) | RZV: 43/278  PLB: 17/274  RR: 2.49 (1.46-4.26) | RZV: 134/283  PLB: 128/279  RR: 1.03 (0.86-1.23) | RZV: 25/283  PLB: 28/279  RR: 0.88 (0.53-1.47) | RZV: 66/283  PLB: 82/279 | RZV: 29/283  PLB: 37/279  RR: 0.77 (0.49-1.22) |
| Dagnew, 2020 | ---- | ---- | ---- | ---- | ---- | ---- | RZV: 144/983  PLB: 112/960 | RZV: 50/983  PLB: 63/960  RR: 0.78 (0.54-1.11) |
| Vink, 2019 | RZV: 94/112  PLB: 7/110  RR: 13.19 (6.41-27.13) | RZV: 13/112  PLB: 0/110  RR: 26.52 (1.60-440.69) | RZV: 91/112  PLB: 73/110  RR: 1.22 (1.04-1.44) | RZV: 25/112  PLB: 17/110  RR: 1.44 (0.83-2.52) | RZV: 100/117  PLB: 103/115  RR: 0.95 (0.87-1.05) | RZV: 18/117  PLB: 15/115  RR: 1.18 (0.63-2.23) | RZV: 30/117  PLB: 31/115 | RZV: 12/117  PLB: 11/115  RR: 1.07 (0.49-2.33) |
| Vink, 2020 | RZV: 115/132  PLB: 12/132  RR: 9.58 (5.57-16.50) | RZV: 14/132  PLB: 0/132  RR: 29.00 (1.75-481.15) | RZV: 90/132  PLB: 73/132  RR: 1.23 (1.02-1.49) | RZV: 13/132  PLB: 11/132  RR: 1.18 (0.55-2.54) | RZV: 51/132  PLB: 44/132  RR: 1.16 (0.84-1.60) | RZV: 7/132  PLB: 5/132  RR: 1.40 (0.46-4.30) | RZV: 26/132  PLB: 33/132 | RZV: 1/132  PLB: 1/132  RR: 1.00 (0.06-15.82) |
| Berkovitz, 2014 | ---- | ---- | ---- | ---- | ---- | ---- | RZV: 6/74 PLB: 2/49 | ---- |

Legend: RZV – recombinant zoster vaccine; PLB – placebo; ADRs – adverse drug reactions; RR – risk ratio
